# NK cell function down regulated by HMGB2 through ANGPT1/PI3K/AKT pathway and its effect on esophageal squamous carcinoma cells

**DOI:** 10.3389/fimmu.2025.1666199

**Published:** 2025-11-07

**Authors:** Xiaodi Yin, Huihong Cai, Aohua Zhang, Weihan Zheng, Jiahui Zhao, Jun Ma

**Affiliations:** Department of Clinical Laboratory, The Second Affiliated Hospital of Zhengzhou University, Zhengzhou, China

**Keywords:** ESCC, natural killer cells, HMGB2, CRISPR-Cas9, ANGPT1, PI3K/Akt pathway

## Abstract

Natural killer (NK) cells are crucial for immune defense against tumors, but their function is often impaired in the tumor microenvironment. High mobility group box 2 (HMGB2), a chromatin-associated protein, is implicated in various cancers, yet its role in regulating NK cells, particularly in esophageal squamous cell carcinoma (ESCC), is unclear. We conducted transcriptomic and proteomic analyses of peripheral blood mononuclear cells (PBMCs) from ESCC patients and healthy donors to identify immunoregulatory molecules. Flow cytometry confirmed upregulation of HMGB2 in NK cells from ESCC patients, correlating with advanced tumor stage. Using RNA interference, CRISPR/Cas9, and overexpression methods, we modulated HMGB2 in NK-92 cells and assessed their cytotoxicity against ESCC cells. HMGB2 silencing or knockout enhanced NK cell cytotoxicity, evidenced by increased granzyme B, perforin, IFN-γ, and TNF-α, and higher tumor cell lysis. Conversely, HMGB2 overexpression suppressed these effects. Mechanistically, HMGB2 ablation induced ANGPT1 expression and activated the PI3K/AKT pathway. ANGPT1 knockdown in KO-HMGB2 NK cells reduced PI3K/AKT activation, confirming the involvement of the ANGPT1/PI3K/AKT axis in enhanced NK cell function. These results indicate that HMGB2 inhibits NK cell-mediated anti-tumor immunity in ESCC. HMGB2 depletion enhances NK cell cytotoxicity via the ANGPT1/PI3K/AKT pathway, suggesting its potential as a therapeutic target to improve NK cell-based immunotherapy in ESCC.

## 1 Introduction

Esophageal squamous cell carcinoma (ESCC) is a highly aggressive and fatal malignancy of the digestive system, with a particularly high incidence in East Asia (1). Although recent advances in surgery, radiotherapy, and chemotherapy have been made, the five-year survival rate for ESCC remains dismal, particularly among patients diagnosed at advanced stages (1, 2). The limited efficacy and significant side effects of conventional treatments have prompted an urgent need to explore more effective therapeutic strategies (3, 4). In recent years, tumor immunotherapy has emerged as a promising modality for various solid tumors and has become a key focus of ESCC research (5).

Peripheral blood mononuclear cells (PBMCs) serve as a valuable indicator of systemic immune status, comprising a diverse array of immune effector and regulatory cells. Although NK cells comprise only 5–20% of PBMCs, they are key effector cells in tumor immune defense, capable of recognizing and eliminating tumor cells independently of antigen presentation (6, 7). NK cells exert their anti-tumor effects primarily by secreting perforin, granzyme B, and cytokines such as IFN-γ and TNF-α (8). However, within the immunosuppressive microenvironment of solid tumors, NK cell function is often severely impaired (9, 10). Accordingly, enhancing NK cell activity and overcoming tumor immune evasion have become major areas of current research (11). Recently, CRISPR/Cas9 gene editing has emerged as a precise and versatile tool for enhancing NK cell function, showing great promise in immune cell engineering (12, 13).

HMGB2 is a non-histone DNA-binding protein that participates in chromatin structure regulation and transcriptional control (14). HMGB2 is frequently overexpressed in various cancers and is often associated with poor clinical outcomes (15, 16). However, its specific role in regulating NK cell-mediated immune function remains unclear. Several studies suggest that the HMGB protein family may regulate innate immune responses by interacting with nucleic acids and immune signaling molecules (17, 18). Given the pivotal role of the PI3K/AKT signaling pathway in NK cell activation and cytotoxicity, we hypothesize that HMGB2 may modulate NK cell anti-tumor function via this signaling axis.

In this study, transcriptomic and proteomic profiling of PBMCs from ESCC patients and healthy donors revealed HMGB2 to be significantly upregulated in ESCC. Using RNA interference and CRISPR/Cas9-mediated gene editing in NK-92 cells, we investigated the functional role of HMGB2 in NK cell activity. Our results demonstrate that HMGB2 suppresses NK cell cytotoxicity by downregulating ANGPT1, thereby inhibiting the PI3K/AKT signaling pathway. These findings suggest that HMGB2 functions as a novel negative regulator of NK cell activity and may represent a promising target for enhancing NK cell-based immunotherapy in ESCC.

## 2 Materials and methods

### 2.1 Human specimens

PBMCs were collected from 5 ESCC patients and 5 healthy controls for transcriptome sequencing, with 3 matched pairs randomly selected for iTRAQ (isobaric tags for relative and absolute quantitation) proteomic analysis. Baseline clinicopathological characteristics are summarized in **Supplementary Table 1**. In addition, anticoagulated whole blood samples were collected from 50 pathologically confirmed ESCC patients and 25 healthy controls for flow cytometry analysis. All enrolled patients were newly diagnosed and treatment-naïve, with no prior history of radiotherapy or chemotherapy before blood collection. This study was approved by the Ethics Committee of the Second Affiliated Hospital of Zhengzhou University (Approval No. 2021190), and written informed consent was obtained from all participants. Fresh anticoagulated blood was centrifuged to remove plasma, and an equal volume of PBS was added to the remaining cellular fraction. PBMCs were isolated using a lymphocyte separation solution (Cedarlane, CL5020). One portion of the isolated cells was treated with TRIzol reagent for transcriptomic analysis, while the remaining cells were subjected to iTRAQ-based proteomic profiling. Transcriptome sequencing was conducted by Novogene Co., Ltd. (Beijing, China) using the Illumina HiSeq platform.iTRAQ-based proteomic analysis was carried out by Gene Create Biological Engineering Co., Ltd. (Wuhan, China) using a Thermo Fisher Q Exactive HF-X mass spectrometer coupled with the UltiMate 3000 RSLC nano system. Mass spectrometry data were analyzed using MaxQuant software (version 1.6.17.0).

### 2.2 Cell culture

The human NK cell line NK-92 (ATCC, CRL-2407, USA) was cultured in alpha-MEM supplemented with 12.5% fetal bovine serum (Biological Industries, Israel, catalog no. 04-001-1), 12.5% horse serum (Solarbio, China), and 20 ng/mL recombinant interleukin-2 (IL-2; PeproTech, 200-02). The ESCC cell lines KYSE70, KYSE150, KYSE450, and EC109 were maintained at the Medical Research Center of the Second Affiliated Hospital of Zhengzhou University. Cells were cultured in RPMI-1640 medium supplemented with 10% FBS, 100U/mL penicillin, and 100μg/mL streptomycin. All cells were incubated in a humidified atmosphere at 37°C with 5% CO_2_.

### 2.3 Cell transfection

For gene knockdown experiments, HMGB2-specific small interfering RNA (siRNA) was transfected into NK-92 cells using Lipofectamine 2000 reagent (Invitrogen, USA, 200-02). Plasmids containing ANGPT1 shRNA sequences were constructed and transfected into HMGB2-knockout NK-92 cells using Lipofectamine 3000 reagent (Invitrogen, USA,11668019), following the manufacturer’s instructions. Cells were cultured in Opti-MEM medium for 48 hours post-transfection. The RNA oligonucleotide sequences used in these experiments were as follows: siHMGB2, #1:5’-CCAACAGGCTCAAAGAAGA-3’; #2: 5’- GCAGTCAGCCAAAGATAAA-3’; #3: 5’- GGCAAAAAGTGACAAAGCT-3’. And siRNA negative control was from Ribobio Co. Ltd (Guangzhou, China).

The full-length HMGB2 coding sequence was synthesized and subcloned into the pcDNA3.1 expression vector. Electroporation was performed by transfecting NK-92 cells with 10 μg of either pcDNA3.1 or pcDNA3.1-HMGB2 plasmids. After thorough mixing, electroporation was conducted using the Gene Pulser Xcell™ system (Bio-Rad) under the following parameters: 200 V, 3-millisecond pulse duration, 3-millisecond intervals, repeated for three pulses. Cells were maintained in Opti-MEM medium for 48 hours after electroporation.

### 2.4 Flow cytometry

A tenfold volume of red blood cell lysis buffer was added to 100 μL of anticoagulated whole blood, followed by incubation on ice for 25 minutes. The blood samples were then washed twice with staining buffer (PBS containing 1% FBS). Approximately 3× 10^5^ PBMCs were isolated and stained with CD3-PerCP (Biolegend, USA, 981016), CD56-PE (Caprico Biotechnology, China, 110625), and CD16-APC (Caprico Biotechnology, China, 101445) in staining buffer. Cells were fixed with Fix/Perm buffer (BD Biosciences, USA, 562574) for 40 minutes at 4°C, washed with staining buffer, and subsequently permeabilized with Perm/Wash buffer (BD Biosciences, USA, 562574). HMGB2 was stained using a rabbit monoclonal antibody (Abcam, ab133540) for 40 minutes at 4°C. Following washing with staining buffer, cells were incubated with FITC-conjugated donkey anti-rabbit IgG secondary antibody (Biolegend, 406403) for 20 minutes. Isotype controls (BioLegend, USA) were used at a dilution of 1:50. Flow cytometry (BeamCyte, China) was performed to evaluate the mean fluorescence intensity and positive expression rate of HMGB2, gated on CD3^+^CD56^+^CD16^+^lymphocyte populations. A total of 2 × 10^4^ lymphocytes were collected for each sample. The culture supernatant from NK cells was collected to measure lymphokines, including TNF-α and IFN-γ, using the Th1/Th2 Subset Detection Kit (Cell-genebio, China, No. 20180030). Samples were analyzed by flow cytometry (BeamCyte, China) and the data were processed using FCAP Array software (BD Biosciences, USA).

The fluorescence channel configuration was as follows: lymphocytes were gated using FSC-H *vs*. SSC-H, single cells by FSC-A *vs*. FSC-H, CD3^-^CD56^+^ NK cells by CD3-PerCP *vs*. CD56-PE, CD3^-^CD16^+^ NK cells by CD3-PerCP *vs*. CD16-APC, and HMGB2^+^ events in NK cell subsets by FITC *vs*. SSC-H. Thresholds were set at 30,000 events to exclude debris, and in most experiments 3–5 × 10^4^ lymphocytes were recorded to ensure robust analysis. Because HMGB2 was highly expressed in NK cell subsets of ESCC patients, fluorescence-minus-one (FMO) controls were not applied; instead, gating positions were determined based on isotype and single-staining controls, which allowed reliable discrimination of positive and negative populations. The gating strategy for flow cytometry is illustrated in **Supplementary Figure S1**.

### 2.5 Real-time PCR

Total RNA was extracted and reverse-transcribed into cDNA using the HiScript^®^ IIQ RT SuperMix for qPCR (Vazyme, China, R222‐01). Quantitative qRT-PCR was performed using FastStart Universal SYBR Green Master (Roche, Switzerland; Cat. No. 04913914001). Primer sequences used in the experiments are listed in **Supplementary Table S2.**

### 2.6 Western blotting

NK-92 cells were lysed in RIPA buffer (Solarbio, Beijing, catalog no. R0020) supplemented with 1% phosphatase and protease inhibitors. The lysates were incubated on ice and quantified using a BCA protein assay kit (Beyotime, Shanghai, China, catalog no. P0012). Protein samples were then mixed with 5× loading buffer (4:1, v/v), followed by boiling at 100 °C for 10 minutes. Proteins were separated by 10% SDS–PAGE and transferred onto PVDF membranes (Millipore, USA, catalog no. IPVH00010). The membranes were incubated with primary antibodies overnight at 4°C. On the following day, the membranes were incubated with HRP-conjugated anti-mouse or anti-rabbit IgG secondary antibodies for 1 hour. Protein bands were visualized using enhanced chemiluminescence (ECL) on a Thermo Fisher iBright 1500 imaging system. The following antibodies were used: anti-HMGB2 (rabbit mAb, Abcam, ab133540, 1:1000), anti-β-actin (mouse mAb, Santa Cruz, sc-81178,1:2000), anti-GAPDH (rabbit mAb, Cell Signaling Technology, 2118S, 1:1000), PI3K/AKT signaling pathway (rabbit mAb, ab283852, 1:1000), pi3k (rabbit mAb, ab283852, 1:1000), anti-ANGPT1(rabbit mAb, Zenbio, ab133540, 1:1000) and HRP-conjugated goat anti-mouse or anti-rabbit IgG (Zenbio, China, catalog no. 511103, 1:2000).

### 2.7 Enzyme−linked immunosorbent assay

NK-92 cells transfected with si-HMGB2, OE-HMGB2, or KO-HMGB2 were co-cultured with KYSE450 cells at an effector-to-target (E:T) ratio of 1:1 in Opti-MEM medium for 12 hours. After centrifugation, the culture supernatant was collected, and the concentrations of granzyme B and perforin 1 were measured using the Human Granzyme B ELISA Kit (E-EL-H1617c, Elabscience, Beijing, China) and Human Perforin 1 ELISA Kit (E-EL-H1123c, Elabscience), respectively, according to the manufacturer’s instructions.

### 2.8 CRISPR/Cas9-mediated HMGB2 gene deletion

NK-92 cells were washed twice with DPBS and resuspended in PBS, followed by cell counting. A total of 1×10^6^ cells were transferred to a 1.5 mL microcentrifuge tube and centrifuged to obtain a cell pellet. Ribonucleoprotein (RNP) complexes were prepared by mixing synthetic gRNA and Cas9 protein at a 5:1 molar ratio. The mixture was incubated at room temperature for 10 minutes to ensure complex stability. Nucleofection was performed using the Neon™ Transfection System (Cat# MPK5000, Thermo Fisher Scientific). Immediately after electroporation, transfected cells were transferred to culture dishes and incubated at 37°C with 5% CO_2_ for recovery. After 24 hours, transfection efficiency and cell viability were assessed. Single-cell sorting was carried out using the DispenCell™ Single-Cell Dispenser (Molecular Devices), and individual transfected cells were seeded into 96-well plates. Monoclonal populations were expanded, and clones with stable growth were selected and validated for HMGB2 gene knockout efficiency by qPCR, Sanger sequencing, and Western blotting. Clones exhibiting 100% HMGB2 knockout were cryopreserved and expanded for subsequent experiments. The sgRNA target sequences were designed using the CRISPOR online tool (http://crispor.tefor.net). CRISPR/Cas9-mediated HMGB2 gene deletion was performed as described above. To further assess the specificity of genome editing, potential off-target sites of the selected sgRNAs were computationally predicted using the CRISPOR online tool. The gRNA sequences used in this study were:

gRNA-A1 CTCGTCAAGTTGCCGTGGCG.
gRNA-A2 GCTAGCATTTGGTAGAGGTA.

### 2.9 RNA sequencing and bioinformatics analysis

Total RNA was extracted from KO-HMGB2 and wild-type NK-92 cells using RNA lysis reagent under RNase-free conditions. RNA quality was assessed, and libraries were constructed for high-throughput sequencing using the Illumina platform. Raw sequencing data were processed with FastQC for quality control and Trim Galore to remove low-quality reads and adapters. Clean reads were aligned to the human reference genome using STAR, and gene expression was quantified using FPKM and TPM values by Sangon Biotech Co. Ltd. (Shanghai, China).

Differentially expressed genes (DEGs) were identified between KO-HMGB2 and wild-type NK cells. Functional enrichment analysis of DEGs was performed using the KEGG database, with pathways considered significant at *p* < 0.05. Enriched pathways and core genes were analyzed to elucidate the molecular mechanisms underlying HMGB2-mediated regulation of NK cell function.

### 2.10 Cytotoxicity assay

Four ESCC cell lines—KYSE70, KYSE150, KYSE450, and EC109—were used as target cells (T). NK-92, si-HMGB2 NK-92, OE-HMGB2 NK-92 and KO-HMGB2 NK-92 lines served as effector cells (E). ESCC target cells were seeded in triplicate into 96-well plates at a density of 1 × 10^4^ cells per well. After 6 hours of adhesion, NK cells were added at effector-to-target (E:T) ratios of 1:1, 2:1, 5:1 and 10:1. The co-culture was maintained in complete ESCC culture medium. Cell viability was assessed using the Cell Counting Kit-8 (CCK-8) assay and absorbance was measured at 450 nm. Proliferation rates of ESCC cells were calculated at 0, 24, and 48 hours post-co-culture. The cytotoxicity of NK-92 cells at each E:T ratio was calculated using the following formula:


$$\begin{array}{c} \text{Cytotoxicity }\left(\%\right)\;\\ =\;({\text{V}}_{\text{OD}}\text{ of ESCC monoculture }-{\text{ V}}_{\text{OD}}\text{ of co}\\ -\text{culture})/\;{\text{V}}_{\text{OD}}\text{ of ESCC monoculture }\times \;100\% \end{array}$$

### 2.11 Statistical analysis

GraphPad Prism (version 10.0, GraphPad Software, CA) was used for statistical analysis. FlowJo software (BD Biosciences, USA) and FCAP Array software were used to analyze and visualize flow cytometry data. P values less than 0.05 were considered statistically significant. All error bars represent the standard deviation (SD) and are presented as mean ± SD.

For normally distributed data with equal variances, unpaired Student’s t-tests were used for two-group comparisons, while the Mann–Whitney U test was applied for non-normally distributed data. For comparisons involving more than two groups (e.g. different effector-to-target ratios in co-culture assays), one-way ANOVA with Tukey’s *post hoc* test was performed. When data did not meet normality assumptions, Kruskal–Wallis tests followed by Dunn’s *post hoc* tests were applied.

In the clinical correlation analysis, receiver operating characteristic (ROC) curve analysis was performed to evaluate the ability of HMGB2 expression in CD3^-^CD56^+^ NK cells to discriminate between early-stage (I–IIA) and advanced-stage (IIB–IV) ESCC patients. The optimal cut-off value was determined using Youden’s index, and the corresponding sensitivity and specificity were calculated.

## 3 Results

### 3.1 HMGB2 and other differential mRNAs/proteins expressed in PBMCs from ESCC patients and healthy individuals

**Figure 1:**
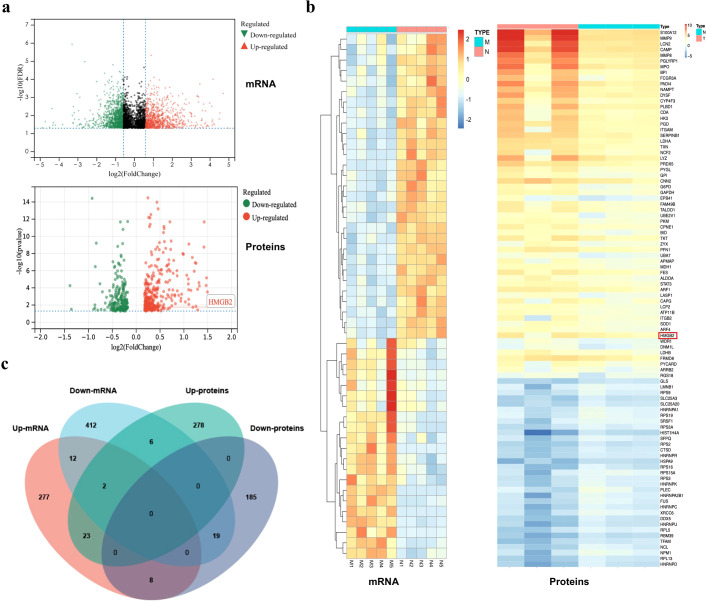
Differential expression of mRNAs and proteins in PBMCs between ESCC patients and healthy individuals. **(a)** Volcano plots showing 3,187 mRNAs and 521 proteins. **(b)** Heatmap of the top 50 differentially expressed mRNAs and top 94 differentially expressed proteins (T/M represents ESCC patients, N represents healthy subjects). **(c)** Venn diagram illustrating the consistent changes in mRNA and protein expression.

A total of 3,187 differentially expressed genes were identified between healthy individuals and ESCC patients (**Figure 1**). Among them, 322 genes were significantly upregulated and 451 were significantly downregulated (*p* < 0.01). Mass spectrometry (MS) identified 521 differentially expressed proteins (**Figure 1a**). The top 50 mRNAs and top 94 proteins (absolute fold change > 1.5) were selected for hierarchical clustering analysis (**Figure 1b**). Combined analysis of transcriptome sequencing and MS data identified 42 genes (including both mRNAs and proteins) showing consistent expression changes, among which 23 were upregulated and 19 downregulated (**Figure 1c**, **Table 1**). HMGB2 emerged as a critical candidate due to its marked upregulation.

**Table 1:** Consistent changes in differentially expressed protein and mRNA.

Protein-Up	ITGA2B, MMP9, PGLYRP1, PYGL, PADI4, DYSF, BPI, SERPINB1, S100A12, ITGB2, PLBD1, TKT, ZYX, **HMGB2**, ITGAM, NBEAL2, APMAP, LSP1, CAPN1, MYO1F, ARHGDIA, GPI, FERMT3
Protein-Down	HNRNPA3, BUB3, FUS, HNRNPK, HNRNPA1, NPM1, HNRNPH1, ATM, RBM39, DHX9, TFAM, RPS3A, RPS3, HSPA9, RPL5, SLC25A3, ANXA2, HNRNPC, HMGB1

HMGB2 is a highly expressed differential protein.

**Figure 2:**
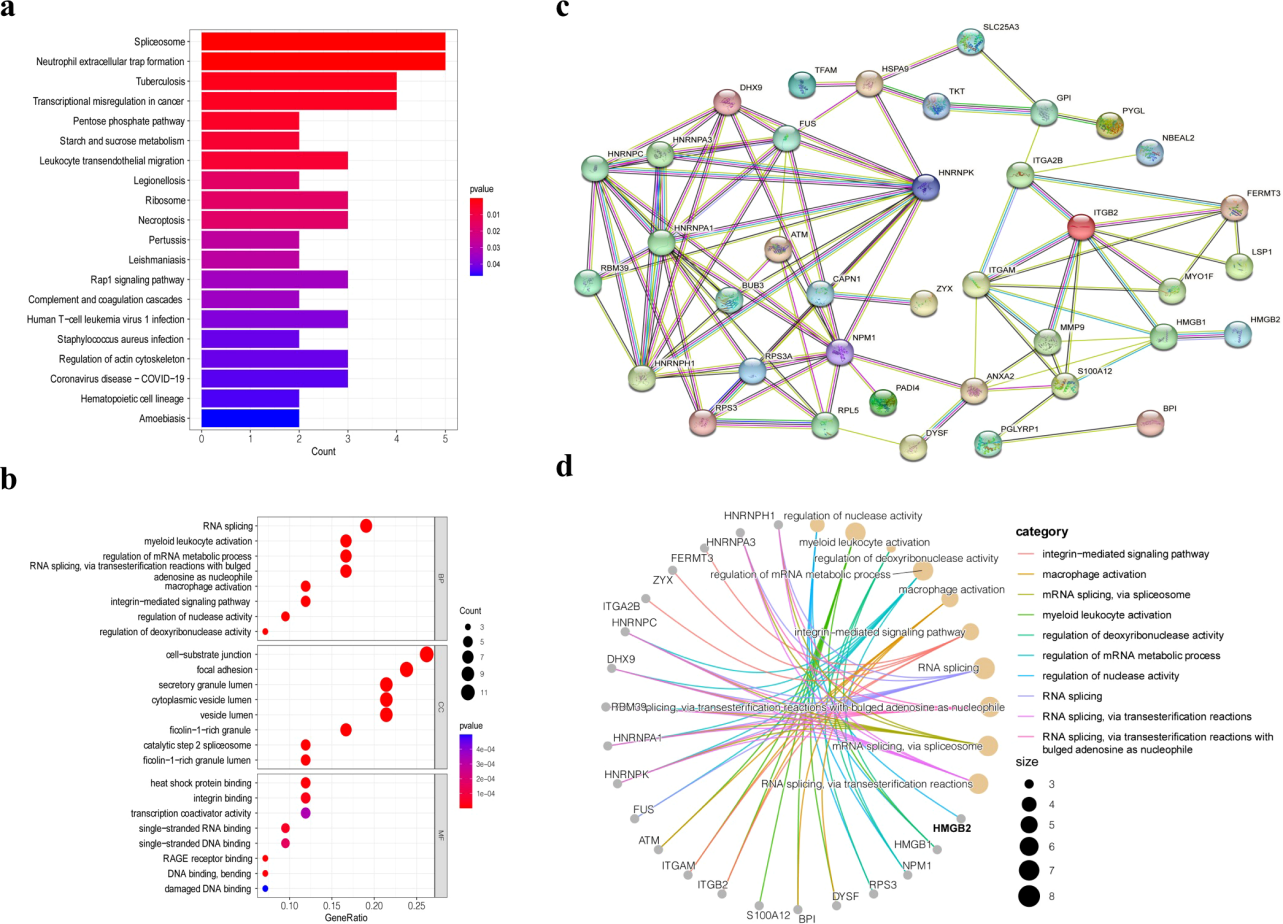
Bioinformatics analysis of 42 differentially expressed genes. **(a)** KEGG pathway analysis of 42 differentially expressed genes. **(b)** GO enrichment analysis of the differentially expressed genes. **(c)** PPI network analysis of the 42 differentially expressed proteins. **(d)** GO pathway network of the 42 differentially expressed proteins.

KEGG and GO enrichment analyses of the 42 genes are shown in **Figure 2**. KEGG pathway analysis revealed that these genes were primarily enriched in spliceosome, neutrophil extracellular trap formation, tuberculosis, and transcriptional misregulation in cancer (**Figure 2a**). GO analysis revealed that in the Biological Process (BP) category, enriched terms included RNA splicing, myeloid leukocyte activation, and regulation of mRNA metabolic processes. In the Cellular Component (CC) category, the enriched terms were cell-substrate junction, focal adhesion, and secretory granule lumen, while the Molecular Function (MF) category included heat shock protein binding, integrin binding, and transcription coactivator activity (**Figure 2b**).

Protein–protein interaction (PPI) network analysis was also performed for the 42 proteins and for HMGB2 specifically (**Figures 2c, d**). HMGB2 showed close association with HMGB1, a member of the same protein family, and was directly linked to granzyme A. Additionally, HMGB2 was found to participate in the regulation of nuclease activity and contribute to transcriptional dysregulation in PBMCs from ESCC patients. These findings highlight the pivotal role of HMGB2 in the pathological alterations observed in PBMCs.

### 3.2 HMGB2 is highly expressed in peripheral blood NK subgroups of ESCC patients and correlated with primary tumor stage

**Figure 3:**
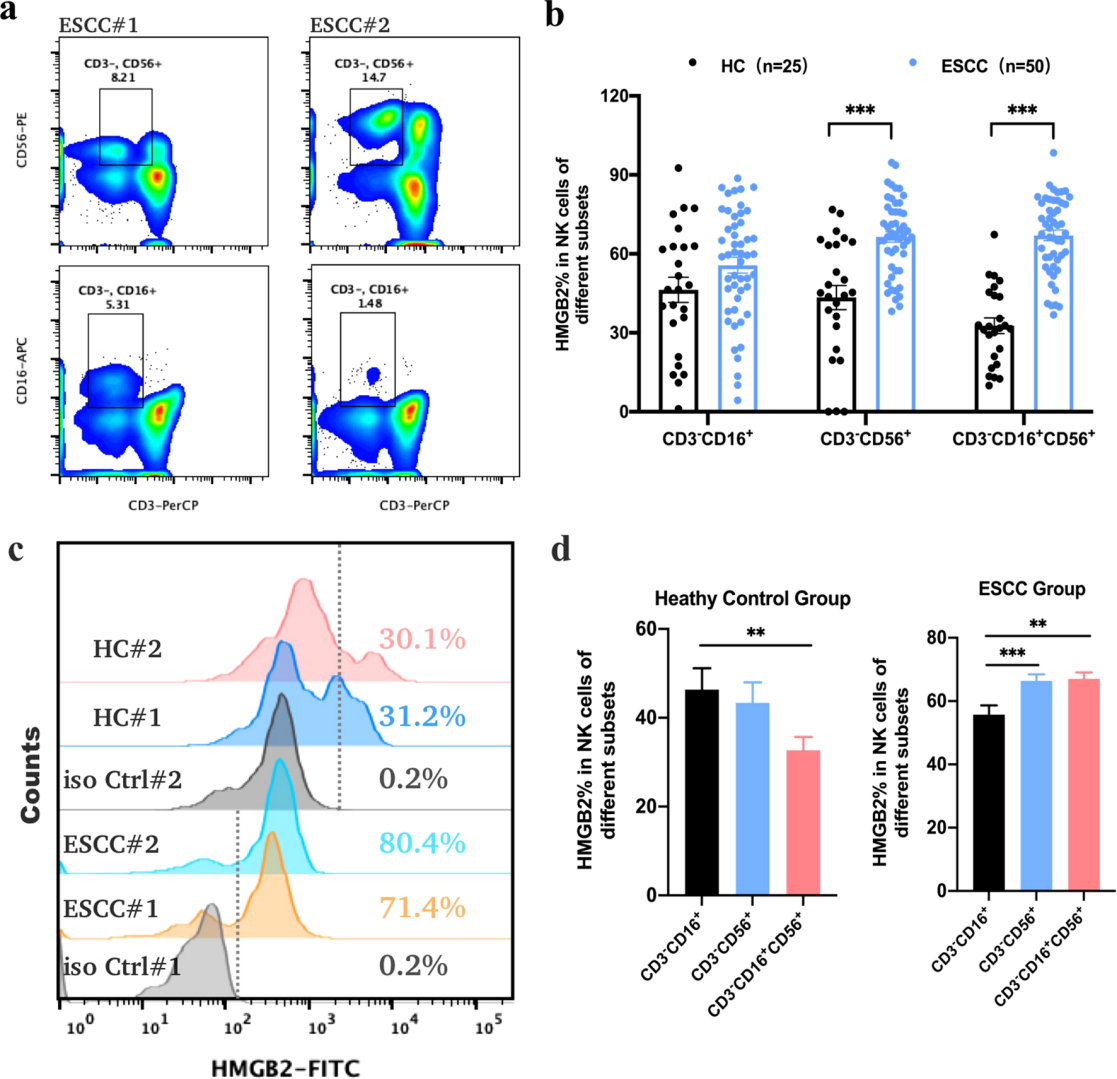
Expression of HMGB2 protein in NK cells as determined by flow cytometry. **(a)** Representative dot plots showing the gating strategy for CD3^-^CD56^+^ and CD3^-^CD16^+^ NK cell populations from the peripheral blood of ESCC patients. **(b)** The positive expression rates of HMGB2 in different NK cell subsets (CD3^-^CD16^+^, CD3^-^CD56^+^, and CD3^-^CD16^+^CD56^+^) from healthy controls (HC, n = 25) and ESCC patients (n = 50). **(c)** Overlaid histograms illustrating HMGB2 expression in NK cells from representative ESCC patients and healthy controls, with fluorescence distributions of isotype controls shown for comparison. **(d)** Bar plots summarizing HMGB2 expression among different NK cell subsets in the HC and ESCC groups. Statistical significance was determined using one-way ANOVA with Tukey’s *post hoc* test for normally distributed data, or Kruskal–Wallis tests followed by Dunn’s *post hoc* tests for non-normally distributed data. *p < 0.05, **p < 0.01, ***p < 0.001. Error bars represent the mean ± SD.

Based on bioinformatic analyses, the HMGB2 gene was selected for further validation in PBMCs from ESCC patients using flow cytometry. HMGB2 protein was highly expressed in T cells, B cells, and NK cells in peripheral blood (**Supplementary Figure S2a**). In this study, 50 newly diagnosed ESCC patients from the Second Affiliated Hospital of Zhengzhou University and 25 healthy controls were enrolled. HMGB2 expression in NK cells was significantly higher in ESCC patients than in healthy individuals (**Figure 3**). Specifically, the HMGB2-positive rates in CD3^-^CD56^+^ and CD3^-^CD16^+^CD56^+^ NK cells in ESCC patients were 66.45 ± 2.03% (*p* < 0.001) and 67.02 ± 2.05% (*p* < 0.001), respectively—both significantly higher than in healthy controls. Although the HMGB2-positive rate in CD3^-^CD16^+^ NK cells showed an upward trend in ESCC patients (55.69 ± 2.99%), the difference compared to healthy controls was not statistically significant (*p* = 0.088) (**Figures 3a, b**). In both healthy controls (HC) and ESCC patient subgroups, HMGB2 expression varied significantly among different NK cell subsets. In the HC group, the HMGB2-positive rate in CD3^-^CD16^+^ NK cells was significantly higher than that in the other two subsets and showed a notable difference compared to CD3^-^CD16^+^CD56^+^ NK cells (*p* < 0.01). In the ESCC group, CD3^-^CD56^+^ NK cells (*p* < 0.001) and CD3^-^CD16^+^CD56^+^ NK cells (*p* < 0.01) showed significantly higher HMGB2-positive rates compared to CD3^-^CD16^+^ NK cells, indicating marked upregulation of HMGB2 in these NK subsets in ESCC patients(**Figure 3c**).

Furthermore, we compared the proportions of various NK cell subsets in PBMCs between ESCC patients and healthy individuals. In ESCC patients, the proportions of CD3^-^CD16^+^ NK cells (8.81 ± 0.73%, *p* = 0.042) and CD3^-^CD16^+^CD56^+^ NK cells (4.76 ± 0.67%, *p* = 0.012) were significantly reduced, whereas the proportion of CD3^-^CD56^+^ NK cells was significantly increased to 14.18 ± 1.02% (*p* = 0.016), as shown in **Supplementary Figure S2b**. These findings suggest a significant alteration in the distribution of NK cell subsets in the peripheral blood of ESCC patients, particularly a marked increase in CD3^-^CD56^+^ NK cells, which may play a critical role in antitumor immune responses. Further investigation into CD3^-^CD56^+^ NK cells as potential biomarkers or therapeutic targets for ESCC is warranted.

To explore the association between HMGB2 expression in CD3^-^CD56^+^ NK cells and the clinicopathological characteristics of ESCC patients, we analyzed their clinical data. The analysis revealed no significant correlation between HMGB2 expression and sex or tumor size, although a weak association with age was noted. Specifically, the HMGB2-positive rate in CD3^-^CD56^+^ NK cells was higher in patients younger than 60 years, although the difference did not reach statistical significance (*p* = 0.061). The HMGB2-positive rate was 63.31 ± 2.77% in Stage I–IIA patients and increased to 75.93 ± 4.18% in Stage IIB–IV patients. The HMGB2-positive rate in Stage IIB–IV patients was 5.67% to 19.57% higher than that in Stage I–IIA patients, indicating a significant positive correlation between HMGB2 expression in NK cells and primary tumor stage (*p* = 0.041), as shown in **Table 2**. Furthermore, ROC curve analysis demonstrated that HMGB2 expression in CD3^-^CD56^+^ NK cells could effectively distinguish early-stage from advanced-stage ESCC patients, with an AUC of 0.82 (95% CI: 0.56–1.00, *p* < 0.01). The optimal cut-off value was 63.2%, yielding a sensitivity of 100% and a specificity of 57.1%.

**Table 2:** Clinicopathological features and correlation of HMGB2 expression in NK cells of PBMCs.

Characteristics	ESCC(n)	HMGB2 (mean ± SEM%)	*P* value
Age(year)			
<60	13	71.47 ± 2.41	0.061
≥60	37	64.68 ± 2.56	
Gender			
Female	13	67.88 ± 4.27	0.680
male	37	65.95 ± 2.32	
Tumor size(cm)			
≤4.0	18	68.86 ± 3.38	0.667
>4.0	15	71.01 ± 3.59	
Pathological N			
N0	19	71.52 ± 2.96	0.939
N1	7	71.07 ± 5.22	
Pathological T			
T1/T2	10	71.10 ± 3.94	0.946
T3/T4	18	70.76 ± 3.05	
Stage			
I-IIA	28	63.31 ± 2.77	0.041*
IIB-IV	7	75.93 ± 4.18	

*P < 0.05; Unpaired t tests, Mann-Whitney test was used in Age group.

### 3.3 Down-regulation of HMGB2 increases the function of NK cells

**Figure 4:**
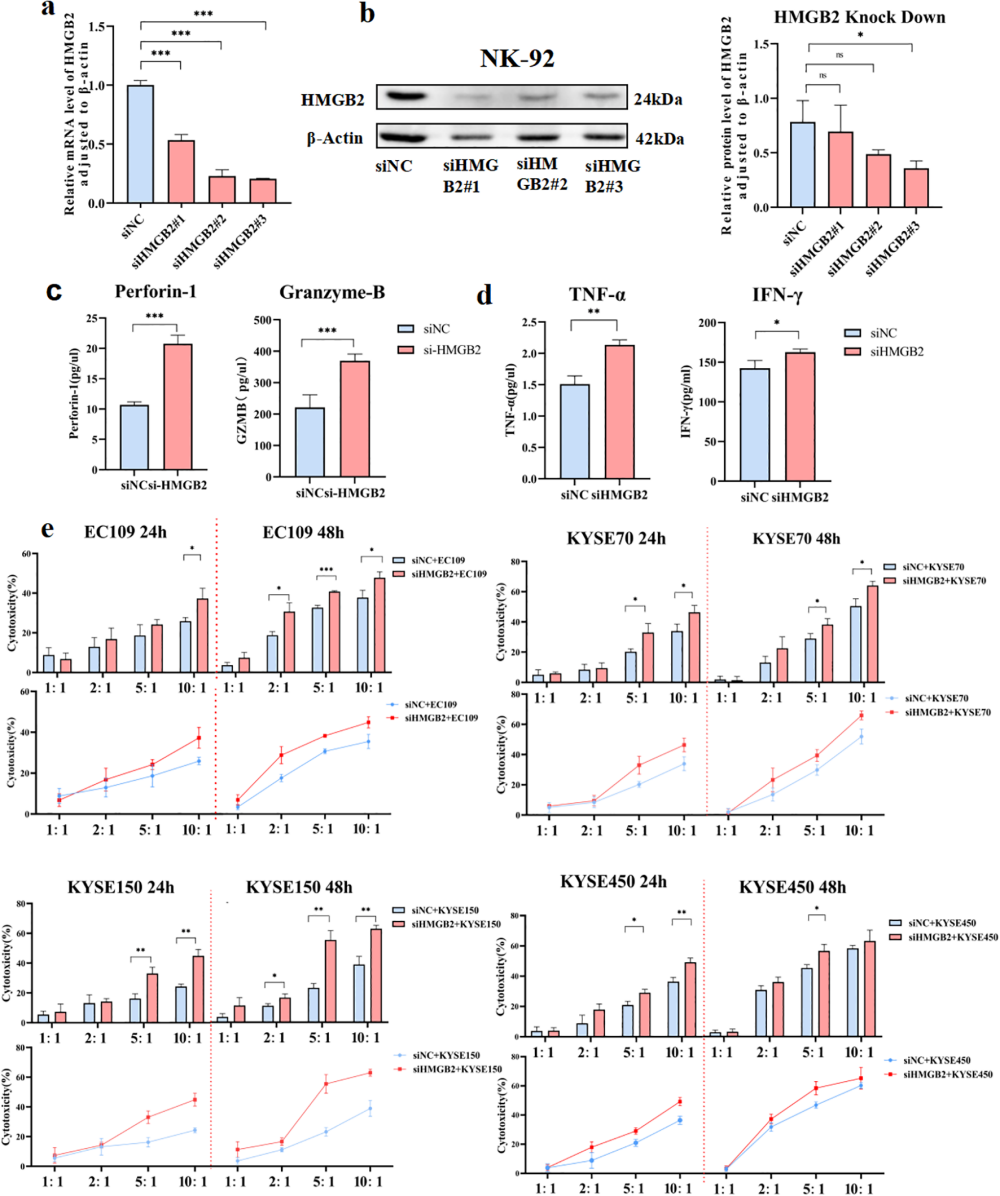
Down-regulation of HMGB2 enhances the function of NK cells. **(a)** qRT-PCR analysis of HMGB2 mRNA levels in NK-92 cells transfected with siNC, siHMGB2 #1, siHMGB2 #2, and siHMGB2 #3. **(b)** Western blot analysis of HMGB2 protein levels in NK-92 cells transfected with siNC or siHMGB2. **(c)** The secretion levels of perforin-1 and granzyme B were measured by ELISA in NK-92 cells after HMGB2 knockdown. **(d)**Cytokine release (TNF-α and IFN-γ) from NK-92 cells with HMGB2 knock down were showed in histogram. **(e)** Cytotoxicity of NK-92 cells with HMGB2 knockdown against various ESCC cell lines (EC109, KYSE70, KYSE150, KYSE450) was assessed at 24h and 48h using a cytotoxicity assay. Statistical significance was determined using one-way ANOVA with Tukey’s *post hoc* test for normally distributed data, or Kruskal–Wallis tests followed by Dunn’s *post hoc* tests for non-normally distributed data. *p < 0.05, **p < 0.01, ***p < 0.001. Error bars represent the mean ± SD.

Given the elevated expression of HMGB2 in NK cells from ESCC patients, we hypothesized that silencing HMGB2 might enhance NK cell function. To test this hypothesis, we employed RNA interference (RNAi) technology (**Figure 4**). Three small interfering RNAs (siRNAs) targeting HMGB2 were designed and transfected into the NK-92 cell line, which is characterized by the expression of CD3^−^CD16^−^ CD45^+^CD56^+^ surface markers (19). NK-92 is a well-established NK cell line with potent antitumor activity and remains the only NK cell line approved for clinical trials to date (20). Numerous clinical trials worldwide have explored the therapeutic potential of genetically modified NK-92 cells for cancer treatment, with several reporting encouraging outcomes. HMGB2 expression was knocked down using the three siRNAs, and qRT-PCR along with Western blot analyses confirmed that siHMGB2#3 achieved the highest gene silencing efficiency in NK-92 cells. Accordingly, siHMGB2#3 was selected for subsequent experiments (**Figures 4a, b**).

The functional activity of NK-92 cells was evaluated by examining the expression of perforin and granzyme B, along with the secretion of lymphokines in the culture supernatant. Notably, the expression levels of perforin and granzyme B—key effector molecules of NK cells—were significantly upregulated (**Figure 4c**). Lymphokine assays further revealed a marked increase in TNF-α and IFN-γ levels in the supernatant following 48 hours of HMGB2 silencing (**Figure 4d**). To further evaluate their functional response, NK-92 cells were co-cultured with ESCC cell lines. The results demonstrated that HMGB2 silencing markedly enhanced NK cell-mediated cytotoxicity against ESCC cells, as indicated by increased tumor cell killing and elevated cytokine production (**Figure 4e**). Collectively, these findings indicate that HMGB2 silencing enhances the cytotoxic functionality of NK-92 cells, as reflected by upregulated expression of cytotoxic proteins, increased cytokine secretion, and improved antitumor activity.

### 3.4 Overexpression of HMGB2 inhibits the function of NK-92 cells

**Figure 5:**
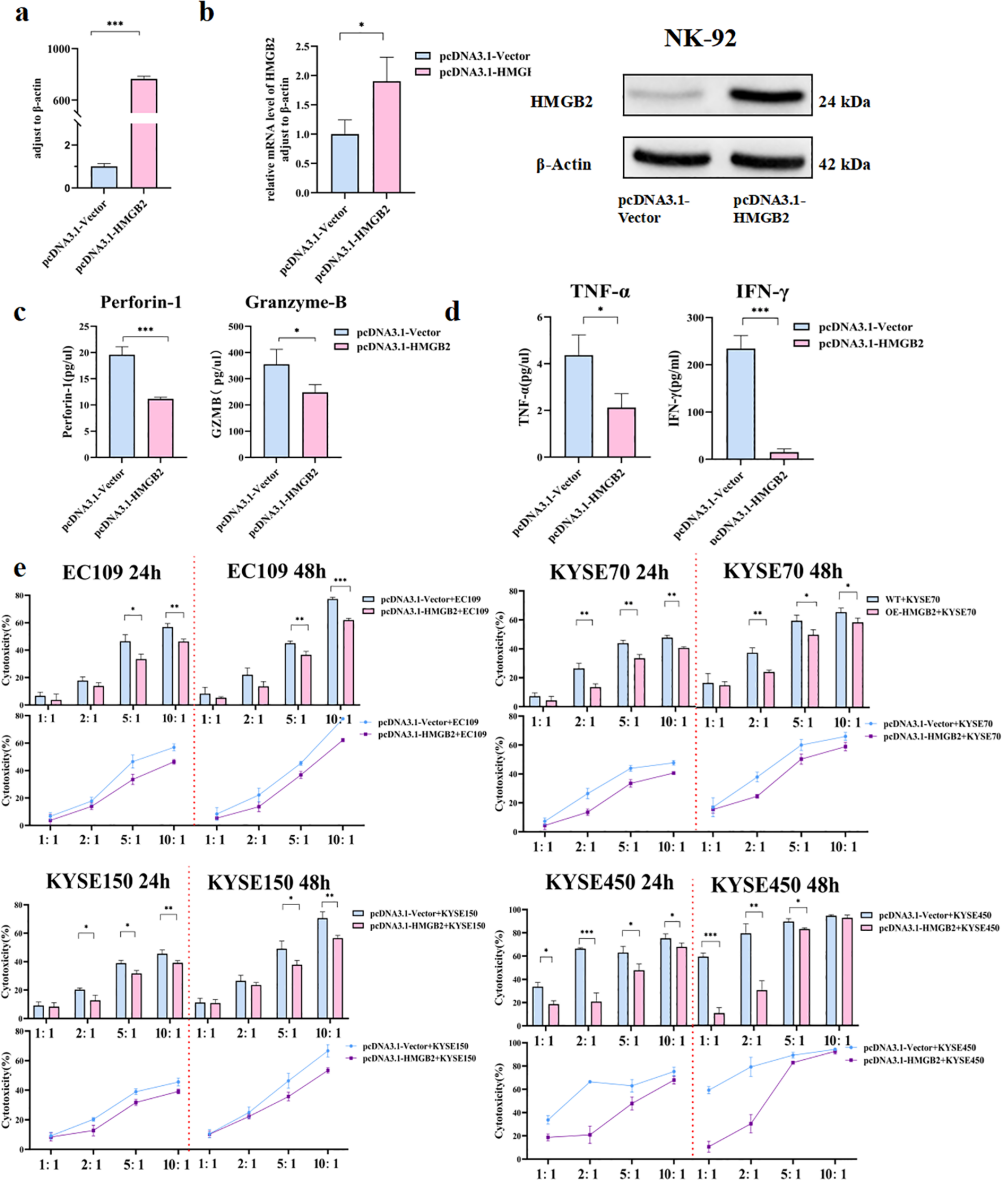
Overexpression of HMGB2 inhibits the function of NK-92 cells. **(a)** qRT-PCR analysis of HMGB2 mRNA levels in NK-92 cells transfected with pcDNA3.1-Vector or pcDNA3.1-HMGB2. **(b)** Western blot analysis of HMGB2 protein expression in NK-92 cells transfected with pcDNA3.1-Vector or pcDNA3.1-HMGB2. **(c)** The secretion levels of perforin-1 and granzyme B were measured by ELISA in NK-92 cells after HMGB2 overexpression. **(d)** Cytokine release (TNF-α and IFN-γ) from NK-92 cells with HMGB2 overexpression was showed in histogram. **(e)** Cytotoxicity of NK-92 cells with HMGB2 overexpression against various ESCC cell lines (EC109, KYSE70, KYSE150, KYSE450) at 24h and 48h was assessed using a cytotoxicity assay. Statistical significance was determined using one-way ANOVA with Tukey’s *post hoc* test for normally distributed data, or Kruskal–Wallis tests followed by Dunn’s *post hoc* tests for non-normally distributed data. *p < 0.05, **p < 0.01, ***p < 0.001. Error bars represent the mean ± SD.

To assess the impact of HMGB2 overexpression on NK cell function, NK-92 cells were transfected with the pcDNA3.1-HMGB2 plasmid to induce HMGB2 expression (**Figure 5**). Successful overexpression was confirmed by both qRT-PCR and Western blot analyses, which demonstrated significantly increased mRNA and protein levels of HMGB2 (**Figures 5a, b**). Following the overexpression of HMGB2, we observed a marked reduction in the expression of key cytotoxic molecules, including perforin and granzyme B, as measured by Elisa (**Figure 5c**). Moreover, flow cytometry analysis revealed a significant decrease in the secretion of pro-inflammatory cytokines, specifically IFN-γ and TNF-α, indicating a suppression of NK cell activation (**Figure 5d**). Additionally, cytotoxicity assays against ESCC cell lines demonstrated that HMGB2 overexpression significantly impaired NK cell-mediated tumor cell killing, with reduced tumor cell lysis observed at multiple effector-to-target ratios (**Figure 5e**). These findings collectively suggest that HMGB2 acts as a negative regulator of NK cell function, and its elevated expression limits the cytotoxic activity and immune response of NK cells.

### 3.5 Construction of NK-92 cell line knocking out HMGB2 using CRISPR/Cas9

Based on the transient results, we decided to construct NK cell lines with complete knockout of HMGB2. In silico off-target analysis was performed using CRISPOR. The results showed that sgA1 exhibited excellent specificity, with MIT and CFD scores of 98 and 99, respectively, and only 14 potential off-target sites, none of which were located in coding exons. In contrast, sgA2 displayed moderate specificity (MIT score 79, CFD score 83) with a larger number of potential off-targets; however, the top-ranked sites were predominantly located in intergenic or intronic regions, suggesting limited functional relevance. Furthermore, no gene editing or phenotypic alterations were detected in the negative control groups (Cas9 only or wild-type NK-92), confirming that the observed genomic modifications were specifically attributable to gRNA-mediated targeting of HMGB2. To confirm the knockout of HMGB2 in NK-92 cells, we performed PCR analysis on the genomic DNA of single-cell clones derived from the stable KO-HMGB2 NK-92 cell line (**Supplementary Figure S3**). Three sets of PCR primers were designed to amplify regions containing the CRISPR-Cas9-induced knockout fragments: Primer 1 targeted the region around the gRNA-A1 sequence; Primer 2 targeted the gRNA-A2 sequence; Primer 3 was designed to amplify sequences flanking the knockout region. Detailed primer sequences are provided in the supplementary table. As shown in **Supplementary Figure S3a**, for Primer 1, no PCR product was detected in the homozygous KO clones, whereas a distinct band was observed at 483 bp in the wild-type cells. Similarly, for Primer 2, no PCR product was detected in the homozygous KO clones, while a clear band at 677 bp was observed in the negative control and wild-type cells (**Supplementary Figure S3b**). For Primer 3, a PCR product was observed at 905 bp in the homozygous KO clones, but the expected band for wild-type cells, at 5336 bp, could not be resolved on the agarose gel due to the large size of the fragment. Consequently, no band was visible for either the homozygous KO or wild-type cells (**Supplementary Figure S3c**).

**Figure 6:**
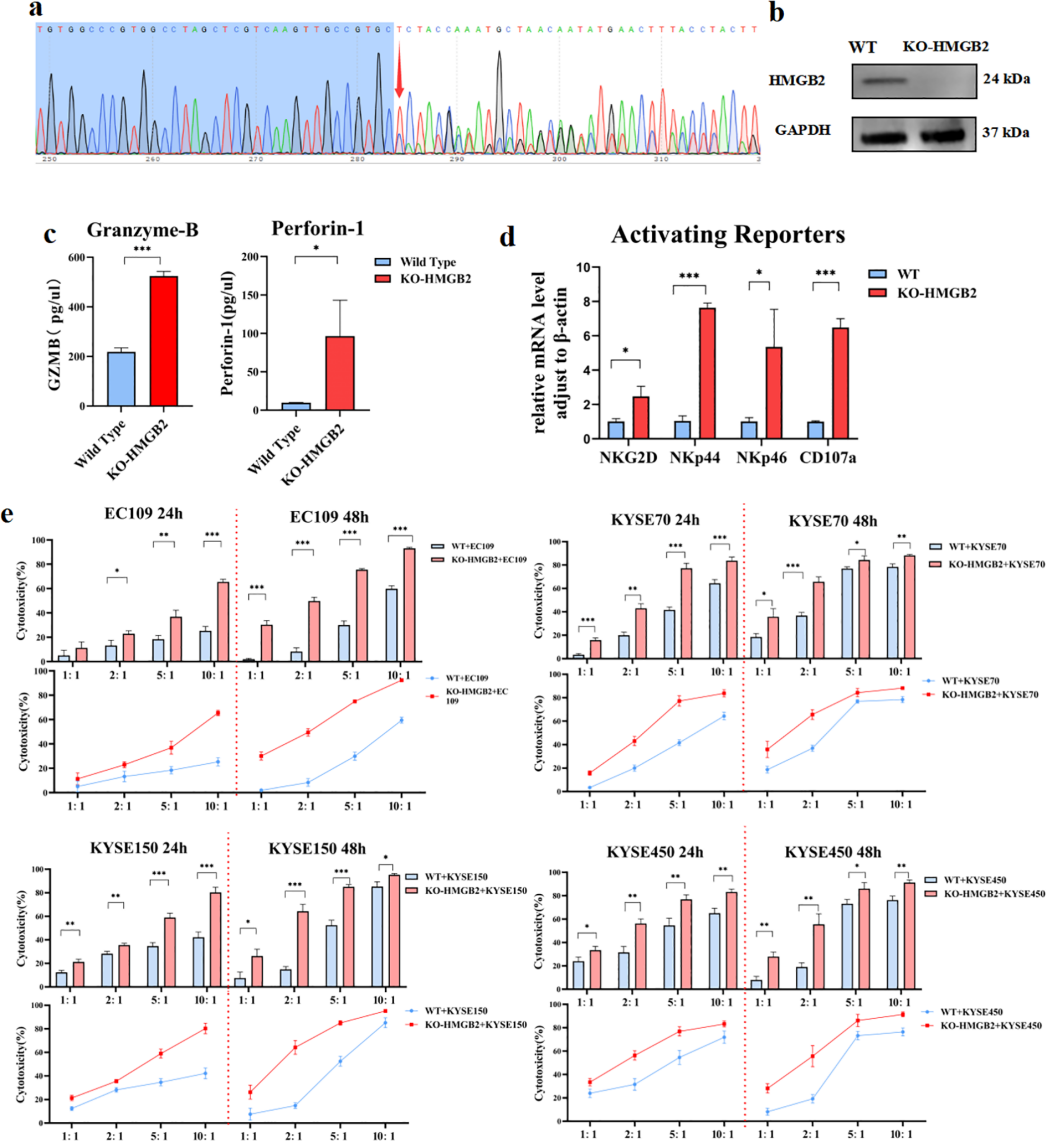
Confirmation of HMGB2 knockout and functional assessment of KO-HMGB2 NK-92 cells. **(a)** Sanger sequencing chromatograms showing two distinct allelic edits in the KO-HMGB2 NK-92 cells. **(b)** Western blot analysis of HMGB2 protein levels in wild-type (WT) and KO-HMGB2 NK-92 cells. **(c)** ELISA analysis of perforin-1 and granzyme B secretion in supernatants collected from KO-HMGB2 and WT NK-92 cells co-cultured with KYSE450 cells for 12 hours. **(d)** qRT-PCR analysis of activating receptors (NKG2D, NKp44, NKp46, and CD107a) in KO-HMGB2 and WT NK-92 cells. **(e)** Cytotoxicity of NK-92 cells with HMGB2 knock out against various ESCC cell lines (EC109, KYSE70, KYSE150, KYSE450) at 24h and 48h was assessed using a cytotoxicity assay. Statistical significance was determined using one-way ANOVA with Tukey’s *post hoc* test for normally distributed data, or Kruskal–Wallis tests followed by Dunn’s *post hoc* tests for non-normally distributed data. *p < 0.05, **p < 0.01, ***p < 0.001. Error bars represent the mean ± SD.

To further validate the gene editing, Sanger sequencing was performed on the HMGB2 gene in the KO-NK-92 cells. two distinct allelic edits in the KO clones: one clone exhibited a 4436bp deletion (ATGTGGCCCGTGGCCTAGCTCGTCAAGTTGCCGTG-del4436bp-CCAAATGCTAGCAGTATGAACTTTACCTACTTCACA), while another clone showed a 4431 bp deletion (ATGTGGCCCGTGGCCTAGCTCGTCAAGTTGCCGTG-del4431bp-CTCTACCAAATGCTAGCAGTATGAACTTTACCTACT), confirming fragment loss or code-shifting mutations in early exons compared to the wild-type sequence and validating the successful knockout of the HMGB2 gene (**Figure 6a**).

Additionally, to assess the efficiency of protein knockout efficiency, Western blot analysis was performed on the KO-HMGB2 NK-92 cells. The results showed that HMGB2 protein expression was completely abolished, confirming that the KO cell line achieved 100% knockout at the protein level (**Figure 6b**). These findings collectively confirm that the HMGB2 gene was successfully knocked out in NK-92 cells at both the genomic and protein levels.

### 3.6 HMGB2-deleted NK-92 cells display stronger functions *in vitro*

NK cell receptors are crucial for tumor cell recognition (21). Compared to wild-type cells, KO-HMGB2 NK-92 cells showed significantly higher mRNA levels of CD107a. Additionally, KO-HMGB2 NK cells exhibited elevated expression of activating receptors, including NKG2D, NKp44, and NKp46 (**Figure 6c**). Supernatants were collected after co-culturing WT and KO-HMGB2 NK-92 cells with KYSE450 cells for 12 hours. Supernatants were collected after co-culturing WT and KO-HMGB2 NK-92 cells with KYSE450 cells for 12 hours. ELISA analysis demonstrated significantly increased secretion of Perforin-1 and Granzyme-B in KO-HMGB2 NK cells in response to tumor stimulation (**Figure 6d**). These findings suggest that HMGB2 knockout in NK cells leads to the upregulation of activating receptors and enhanced secretion of key cytotoxic molecules, collectively contributing to improved NK cells cytotoxicity against tumor cells.

To evaluate the cytotoxicity of KO-HMGB2 NK-92 cells against ESCC cells, tumor cell lysis was assessed after co-culturing NK cells with ESCC cell lines at different E:T ratios for 24 and 48 hours. The results showed a significant increase in the cytotoxic efficiency of KO-HMGB2 NK-92 cells compared to wild-type NK-92 cells across all tested ESCC cell lines. At 24 hours, EC109, KYSE70, KYSE150, and KYSE450 exhibited superior cytotoxicity to wild-type NK cells at all E:T ratios, and the cell killing by KO-HMGB2 NK cells continued to increase with increasing E:T ratios. This trend persisted at 48 hours. When the E:T ratio reached 10:1, the cytotoxic efficiency plateaued due to the limited number of KYSE70, KYSE150, and KYSE450 cells available for lysis. In the co-culture experiments at 24 and 48 hours, KO-HMGB2 NK cells demonstrated consistently higher cytotoxicity than the WT group, and the difference in cytotoxicity became more pronounced with the increasing E:T ratio and extended time (**Figure 6e**). These data suggest that KO-HMGB2 NK-92 cells exhibit superior anti-tumor activity *in vitro*. The role of HMGB2 in regulating NK cell function was further supported, suggesting that HMGB2 may be a potential therapeutic target for enhancing NK cell-mediated anti-tumor immunity.

### 3.7 Knockout HMGB2 enhances PI3K-AKT phosphorylation levels in NK-92 cells by upregulating ANGPT1

**Figure 7:**
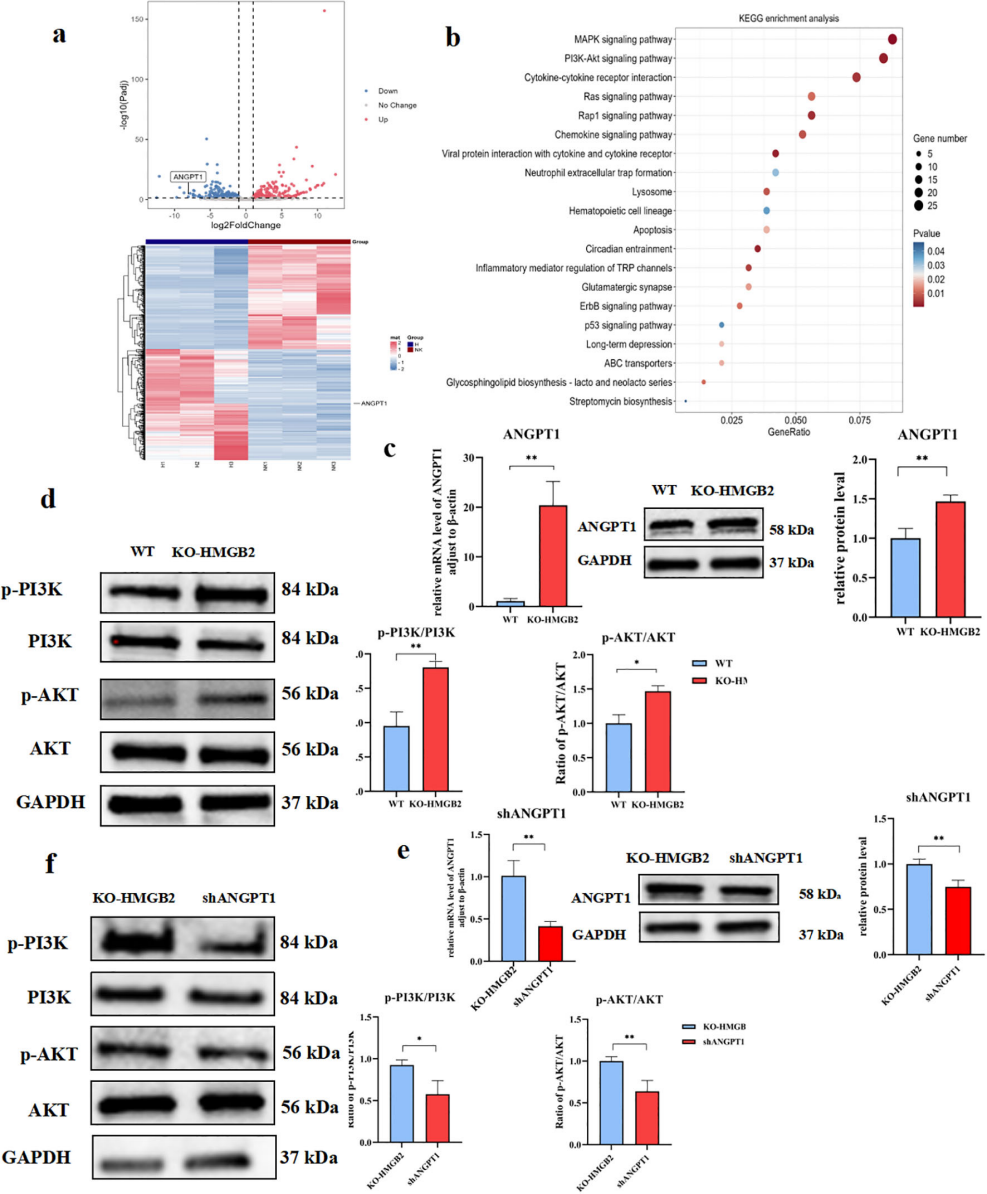
Knockout of HMGB2 enhances PI3K-AKT phosphorylation levels in NK-92 cells by upregulating ANGPT1. **(a)** Differential gene expression analysis between KO-HMGB2 NK cells and wild-type (WT) NK cells. The volcano plot (upper panel) highlights significant changes in gene, while the expressionheatmap (lower panel) displays the expression levels of differentially expressed genes. **(b)** KEGG pathway enrichment analysis of differentially expressed genes in KO-HMGB2 NK cells. **(c)** qRT-PCR and Western blot analysis of ANGPT1 expression in KO-HMGB2 NK cells compared to WT NK cells. **(d)** Western blot analysis of PI3K/AKT signaling in KO-HMGB2 NK cells, The histogram (right panel) shows the quantitative analysis of protein expression. **(e)** qRT-PCR and Western Blot analysis was performed to evaluate ANGPT1 mRNA expression in KO-HMGB2 NK cells following ANGPT1 knockdown. **(f)** Western blot analysis of PI3K/AKT signaling after ANGPT1 knockdown in KO-HMGB2 NK cells, The histogram (right panel) shows the quantification of protein expression. Data are expressed as means ± SD from three independent experiments. Statistical significance was determined by unpaired t-test or one-way ANOVA; *P < 0.05, **P < 0.01.

To further investigate the impact of HMGB2 knockout on the NK cell transcriptome, we conducted high-throughput RNA sequencing comparing KO-HMGB2 NK cells with wild-type NK-92 cells, using |log2FoldChange| > 1 as the screening threshold. Compared to wild-type NK cells, a total of 733 differentially expressed genes were identified in KO-HMGB2 NK cells, including 333 upregulated genes and 400 downregulated genes (**Figure 7a**). Transcriptome analysis revealed that KO-HMGB2 significantly altered the gene expression profile of NK cells, influencing several biological processes and signaling pathways. KEGG pathway enrichment analysis identified several signaling pathways that were significantly differentially expressed in KO-HMGB2 NK cells. Among these, the MAPK signaling pathway, PI3K-Akt signaling pathway, cytokine-cytokine receptor interactions, Ras signaling pathway, and chemokine signaling pathway were upregulated in KO-HMGB2 NK cells, suggesting that these pathways may play a key role in the KO-HMGB2-induced enhancement of NK cell function (**Figure 7b**). ANGPT1 was upregulated in KO-HMGB2 NK cells and was involved in the enrichment of the PI3K-Akt signaling pathway. This suggests that HMGB2 knockout activates the PI3K-Akt pathway by upregulating ANGPT1 expression, thereby enhancing the immune response of NK cells.

To evaluate the impact of KO-HMGB2 on ANGPT1 expression, we first assessed ANGPT1 expression in KO-HMGB2 NK cells and wild-type (WT) NK cells using qRT-PCR and Western blotting. As shown in **Figure 7c**, qRT-PCR analysis revealed that ANGPT1 mRNA levels were significantly higher in KO-HMGB2 NK cells compared to WT NK cells (*p* < 0.01), indicating that HMGB2 knockout upregulated ANGPT1 transcription. Western blotting further confirmed ANGPT1 expression, showing that protein levels significantly elevated in KO-HMGB2 NK cells compared to WT NK cells, consistent with the mRNA results (**Figure 7c**). To investigate PI3K-AKT pathway activation in KO-HMGB2 NK cells, we analyzed the protein expression of p-PI3K, PI3K, p-AKT, and AKT using Western blotting. The results showed a significant increase in the phosphorylation levels of p-PI3K and p-AKT in KO-HMGB2 NK cells. Furthermore, the ratios of phosphorylated PI3K and AKT to total PI3K and AKT were significantly higher in the KO-HMGB2 group compared to WT NK cells (*p* < 0.01), confirming PI3K/AKT pathway activation in KO-HMGB2 NK cells (**Figure 7d**). To further explore the role of ANGPT1 in activating the PI3K-AKT pathway in KO-HMGB2 NK cells, we conducted ANGPT1 knockdown and analyzed the phosphorylation levels of the PI3K-AKT pathway (**Figure 7e**). Western blot analysis showed that ANGPT1 knockdown significantly reduced the protein levels of p-PI3K and p-AKT. Quantitative analysis further supported these findings, indicating that ANGPT1 knockdown inhibited PI3K/AKT pathway activation (**Figure 7f**). While ANGPT1 and p-PI3K/p-AKT levels were elevated in KO-HMGB2 NK cells, ANGPT1 expression was markedly reduced in the shANGPT1 group, accompanied by a significant decrease in p-PI3K and p-AKT phosphorylation. These results validate that HMGB2 knockout in NK cells promotes PI3K/AKT pathway activation through upregulation of ANGPT1.

## 4 Discussion

Esophageal squamous cell carcinoma (ESCC) is a highly aggressive malignancy with extremely limited therapeutic options, especially in advanced stages (22). Despite recent advances in immunotherapy such as immune checkpoint inhibitors and CAR-T cell therapy, the treatment of ESCC still faces great challenges (23–25). In addition, an increasing number of clinical trials are focusing on the use of monoclonal antibodies in combination with chemotherapy/radiotherapy in patients with esophageal squamous carcinoma (ESCC) (26). Meanwhile, peptide vaccine therapies have also been used in clinical trials for ESCC (27, 28). However, these studies generally suffer from low remission rates and high side effects. The limited effectiveness of conventional therapies has prompted the search for superior therapeutic strategies for esophageal cancer, especially immunotherapy (29, 30).

Peripheral blood serves as a window to the body’s immune status, and alterations in the number or function of immune cells in its PBMCs can be detected during tumorigenesis. Relay immune cell therapy, such as LAK (lymphokine-activated killer cells), CIK (cytokine-induced killer cells), and NK cells, have been used in tumor patients for many years (30–33). As important effector cells of the innate immune system, NK cells play a key role in anti-tumor immunity through their cytotoxic effects and cytokine secretion capacity (34). Therefore, strategies to enhance the function of NK cells are expected to provide new breakthroughs in ESCC immunotherapy.

In this study, PBMCs from ESCC patients and healthy individuals were analyzed by combined transcriptomic and proteomic approaches, and HMGB2 was found to be significantly upregulated at both the mRNA and protein levels. Flow cytometry further verified that HMGB2 expression was markedly elevated in T cells, B cells, and particularly in the CD3^-^CD56^+^ NK cell subset of ESCC patients, where its expression level was positively correlated with tumor stage. These findings suggest that HMGB2 may serve as a potential marker for immune dysfunction and tumor progression in ESCC. Nevertheless, a notable limitation of this study is the relatively small sample size used for transcriptomic and proteomic profiling (5 ESCC *vs*. 5 healthy PBMC samples, with iTRAQ performed on 3 paired cases). Such a small cohort inevitably reduces the statistical power of differential expression analysis and may increase the risk of false positives, thereby limiting the generalizability of the omics results. To address this concern, we further validated HMGB2 expression in an extended cohort of 50 ESCC patients and 25 healthy controls using flow cytometry, which provided consistent results and reinforced the reliability of our findings. However, the clinical stage distribution of these 50 ESCC patients was relatively unbalanced, with more early-stage (I–IIA) than advanced-stage (IIB–IV) cases, reflecting the clinical availability of our samples. Despite this limitation, HMGB2 expression remained significantly higher in advanced stages, supporting the robustness of the stage-related association. In addition, paired pre- and post-treatment samples were not available in our current cohort, which prevented us from dynamically evaluating HMGB2 expression during therapy. Even so, larger, multi-center studies will be necessary to further strengthen the robustness of these conclusions and minimize potential bias.

To further explore the role of HMGB2 in NK cell function, we employed siRNA interference and CRISPR/Cas9 gene editing techniques for gene silencing and knockout in NK-92 cells. It is worth mentioning that CRISPR/Cas9 technology has the advantages of high efficiency, high specificity and long-term stable expression in NK cell research (11, 12, 35, 36). In recent years, CRISPR-Cas9 ribonucleoprotein (RNP) complex-mediated gene editing has emerged as a powerful tool in genetic research and immune cell engineering due to its high efficiency, specificity, minimal off-target effects, and independence from exogenous DNA templates. Compared with traditional plasmid- or viral vector-mediated approaches, the RNP method significantly reduces immunogenicity and enhances editing efficiency, making it particularly suitable for primary cells and cell lines such as NK cells, which are typically difficult to transfect (37, 38). We employed the CRISPR/Cas9 RNP approach to establish an NK-92 cell line with complete knockout of HMGB2.In this study, we successfully constructed a stable NK-92 cell line with 100% knockout of the HMGB2 gene, which was validated at both the genomic and protein levels. CRISPR/Cas9 not only provides a reliable model for long-term functional studies, but also provides the technical basis for NK cell engineering transformation and clinical translation.

Functional studies showed that down-regulation of HMGB2, either by RNA interference or CRISPR knockout, significantly enhanced the killing ability of NK cells, as evidenced by elevated expression of perforin and granzyme B, as well as significantly up-regulated secretion levels of key cytokines such as IFN-γ and TNF-α. In contrast, overexpression of HMGB2 inhibited the expression of these effector molecules, suggesting that HMGB2 may act as an immunosuppressive factor in NK cells. Mechanistically, transcriptome and signaling pathway analyses of NK cells after HMGB2 knockdown showed significant activation of the PI3K-AKT signaling pathway, accompanied by upregulation of ANGPT1 expression. Further knockdown experiments confirmed that ANGPT1 is an important regulator of the activation of this pathway, suggesting that HMGB2 may indirectly inhibit the PI3K-AKT signaling pathway by suppressing ANGPT1 expression, leading to a decline in NK cell function. This study clarifies for the first time the key role of the HMGB2-ANGPT1-PI3K/AKT axis in the regulation of NK cell immune function. Nevertheless, the precise mechanism by which HMGB2 regulates ANGPT1 transcription remains unclear. In particular, it is unknown whether HMGB2 directly binds to the ANGPT1 promoter/enhancer to repress its transcription, or acts indirectly through intermediate signaling regulators. As suggested by the reviewers, chromatin immunoprecipitation (ChIP) or promoter reporter assays would provide direct mechanistic evidence. Due to technical limitations, these experiments were not performed in the current study, but they will be the focus of our future work. In an *in vitro* co-culture assay, we further confirmed that HMGB2-deficient NK cells showed enhanced killing ability on a variety of ESCC cell lines, especially in poorly differentiated tumor cells such as KYSE70 and KYSE150. This enhanced effect persisted at different effector ratios and time points, showing good stability and reproducibility, further highlighting its potential clinical value.

From a broader perspective, CRISPR/Cas9 technology has emerged as a key tool for NK cell research and therapeutic development. Recent studies have demonstrated that it is possible to obtain engineered NK cells with sustained expression, enhanced recognition and higher anti-tumor activity, and that the permanent modification advantage of CRISPR over short-lived siRNA interference is particularly suited to the development of ‘off-the-shelf’ NK cell immunotherapies. development (13, 39–42). The KO-HMGB2 NK-92 cell model constructed in this study provides a good theoretical and experimental basis for in-depth research and clinical application in this direction. Despite the strengths of this study, several limitations should be acknowledged. First, the functional validation was conducted exclusively in the NK-92 cell line, which cannot fully recapitulate the biology of primary NK cells from patients or healthy donors. Although NK-92 cells are widely used as a model, validation in primary NK cells will be required to strengthen the translational relevance of our findings. Besides, the present work lacks *in vivo* validation, such as xenograft models of NK cell therapy, which would be important to confirm the antitumor activity of HMGB2-deficient NK cells under physiological conditions. Future studies employing animal models will be necessary to establish the therapeutic relevance of HMGB2 targeting in ESCC. Second, although CRISPR/Cas9 RNP editing has been reported to exhibit high specificity and reduced off-target activity compared with plasmid- or virus-mediated approaches, the possibility of off-target effects cannot be completely excluded. Comprehensive assessments, such as whole-genome sequencing or targeted deep sequencing, will be required in future studies to rigorously evaluate and minimize these risks. Acknowledging these limitations highlights important avenues for further research and strengthens the translational potential of our findings. Furthermore, our study did not evaluate the potential contribution of other immune cell populations in the tumor microenvironment, such as T cells, B cells, or myeloid-derived suppressor cells, which may interact with NK cells and influence the immunological role of HMGB2. In addition, although we extended our analysis to a cohort of 50 ESCC patients, the sample size remains relatively limited, which may reduce statistical power and increase the risk of type II errors, particularly in subgroup analyses. Larger, multi-center cohorts will be required to validate and generalize our conclusions. Another limitation of our study is the lack of survival data for the patient cohort. Although we observed a significant correlation between HMGB2 expression in NK cells and ESCC stage, we were unable to perform survival analyses such as Kaplan–Meier or Cox regression due to the absence of follow-up information. As a result, the prognostic value of HMGB2 expression remains to be determined. Future prospective studies with well-documented patient outcomes will be necessary to clarify whether HMGB2 can serve not only as a stage-related biomarker but also as an independent predictor of patient survival. Interestingly, our findings that HMGB2 suppresses NK cell function contrast with previous reports that HMGB1 enhances NK cell activation and cytotoxicity (43). This apparent antagonism between members of the same HMGB family highlights the complexity of their roles in immune regulation. One possible explanation lies in differences in receptor binding specificity: HMGB1 has been reported to interact with TLR2, TLR4, and RAGE, thereby stimulating NK cell activation through pro-inflammatory signaling, whereas HMGB2 may engage distinct receptors or co-factors that lead to inhibitory effects. Another explanation may be related to subcellular localization. HMGB1 is actively secreted as a damage-associated molecular pattern (DAMP) to boost immune activation, while HMGB2 is more tightly regulated within the nucleus and may indirectly restrain immune cell function. These contrasting modes of action may underlie the differential effects of HMGB1 and HMGB2 on NK cells, and further studies will be required to clarify the molecular determinants that govern their distinct immunological functions.

In summary, we have revealed for the first time that HMGB2 acts as a negative regulator in NK cells, and clarified that it inhibits the PI3K/AKT signaling pathway by suppressing the expression of ANGPT1, leading to the functional limitation of NK cells. From a translational perspective, our findings suggest that HMGB2 may serve as a promising therapeutic target to restore NK cell function in ESCC. Several potential implementation pathways could be envisioned. First, the development of small-molecule inhibitors against HMGB2 may be feasible, drawing on experiences with inhibitors of other nuclear proteins, although challenges remain regarding specificity and delivery. Second, considering that PD-1/PD-L1 immune checkpoint inhibitors are already widely used in ESCC, targeting HMGB2 in combination with checkpoint blockade may exert synergistic effects, thereby further enhancing NK cell–mediated antitumor activity. Finally, integrating HMGB2 knockout into CAR-NK cell platforms may improve the persistence and cytotoxicity of engineered NK cells, thus strengthening the therapeutic potential of adoptive NK cell therapy. These possible strategies highlight the translational relevance of HMGB2 inhibition and warrant further preclinical and clinical investigation. This study not only elucidates the molecular mechanism of NK cell dysfunction, but also provides a theoretical basis for the development of CRISPR-engineered NK cells.HMGB2 is expected to become a novel target for ESCC immunotherapy and provide an innovative direction for clinical NK cell therapy strategies.

## 5 Conclusion

In conclusion, this study identifies HMGB2 as a novel negative regulator of NK cell function in esophageal squamous cell carcinoma (ESCC). Elevated expression of HMGB2 correlates with tumor progression and NK cell dysfunction in ESCC patients, highlighting its potential role as a biomarker for disease staging. Using CRISPR-Cas9 gene editing, we successfully generated HMGB2-knockout NK-92 cells, which exhibited enhanced activating receptor expression, increased secretion of cytotoxic molecules (granzyme B, perforin) and cytokines (IFN-γ, TNF-α), and significantly improved cytotoxic activity against ESCC cell lines. Mechanistically, HMGB2 depletion enhanced NK cell function through activation of the ANGPT1/PI3K/AKT signaling pathway. These findings not only reveal a critical immunoregulatory role of HMGB2 but also provide compelling evidence for targeting HMGB2 to potentiate NK cell-based immunotherapy in ESCC.

## Data Availability

The original contributions presented in the study are included in the article/**Supplementary Material**. Further inquiries can be directed to the corresponding author.
