# Supplementary material for: NK cell function down regulated by HMGB2 through ANGPT1/PI3K/AKT pathway and its effect on esophageal squamous carcinoma cells
Source: Front Immunol. 2025 Nov 7;16:1666199. doi: 10.3389/fimmu.2025.1666199 (PMC12634629; doi:10.3389/fimmu.2025.1666199)
Supplement: Supplementary file 1 [file DataSheet1.pdf]

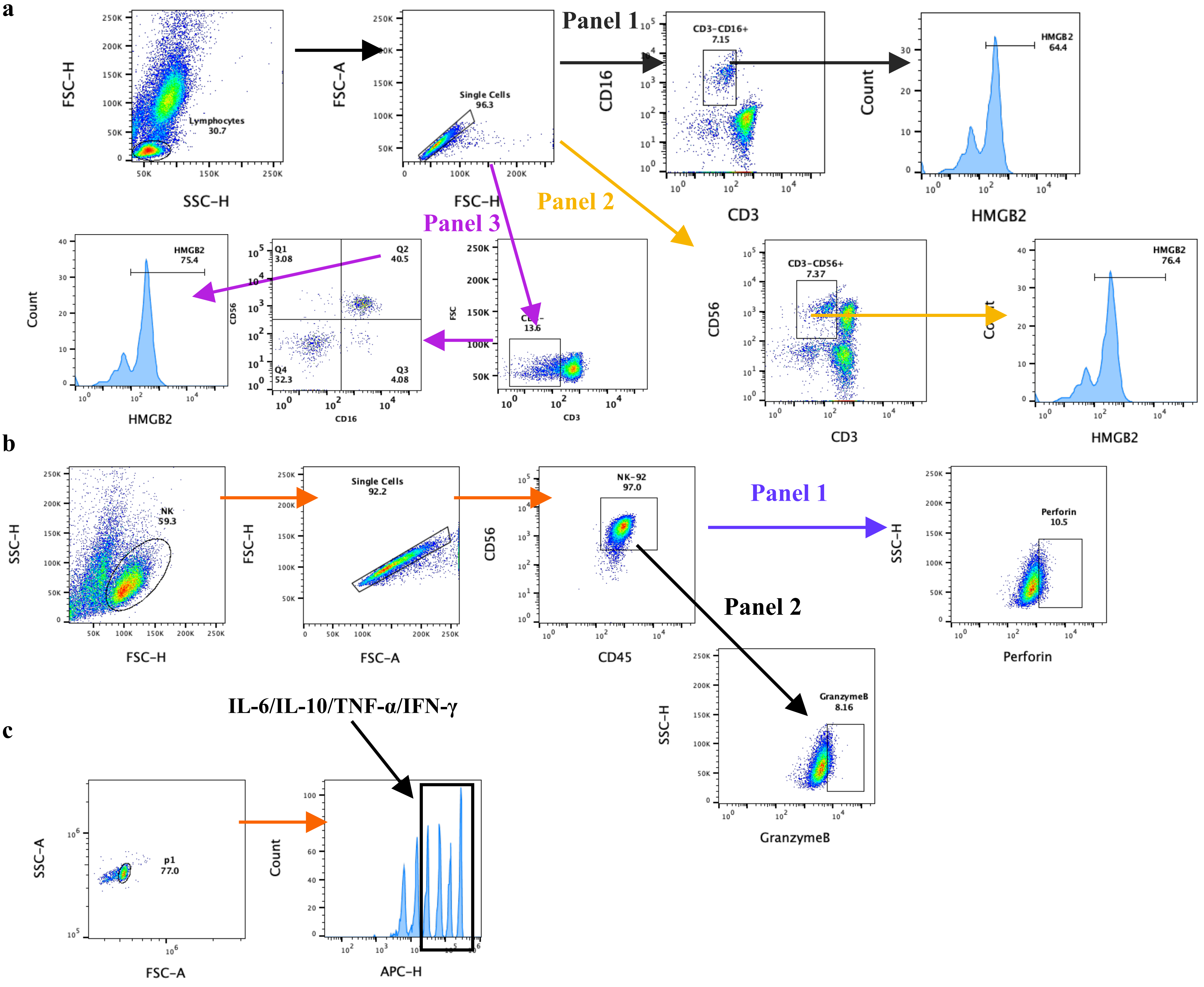

Fig. S1 Flow cytometry gating strategies. **a** Clinical verification of HMGB2 in NK cells subsets; **b** Gating strategies of NK cells function assay; **c** Lymphatic factors gating strategies.

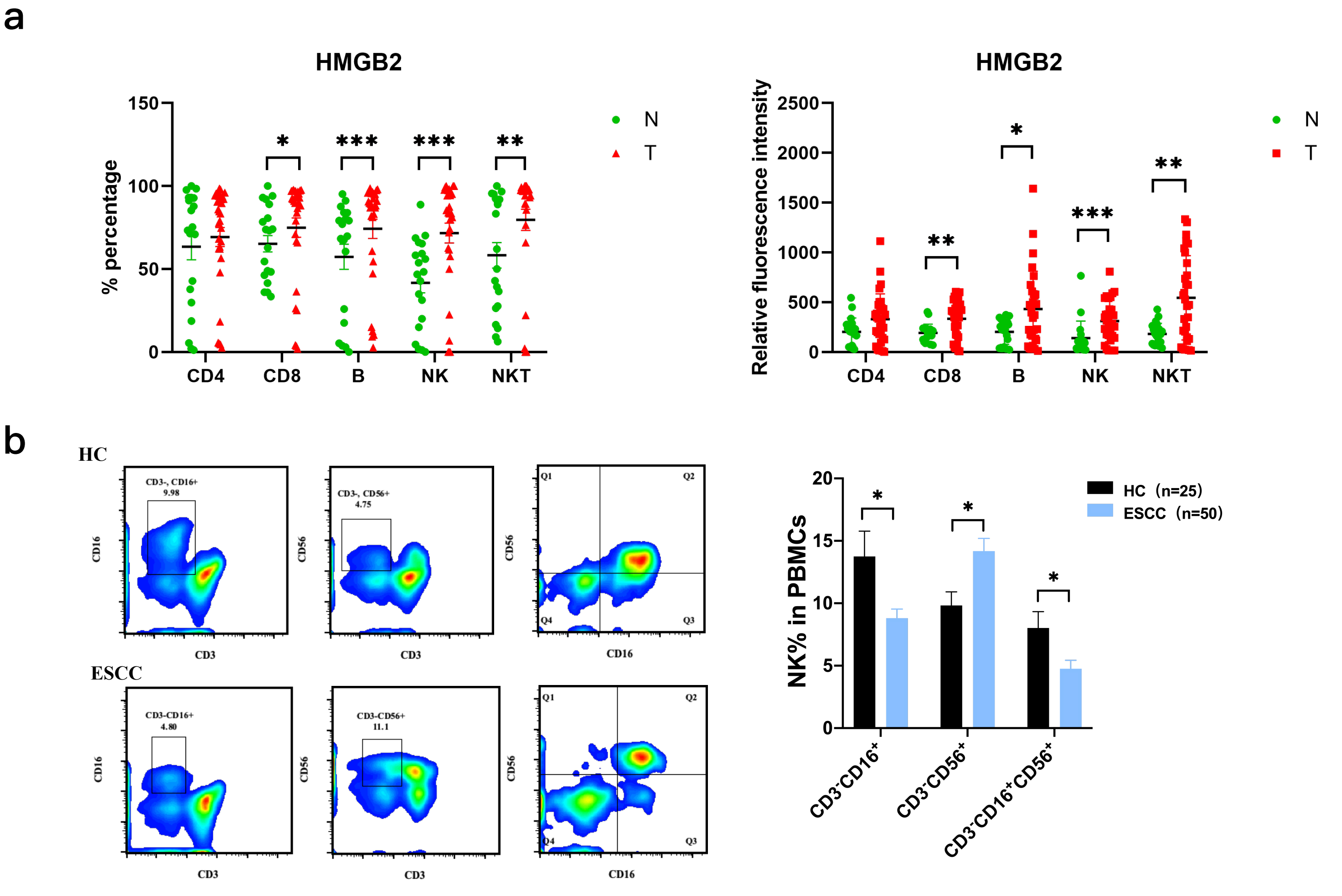

Fig. S2 Clinical verification of HMGB2 in PBMCs subsets; **a** Percentages and MFI of HMGB2 in PBMCs; **b** NK cells% in different subsets.

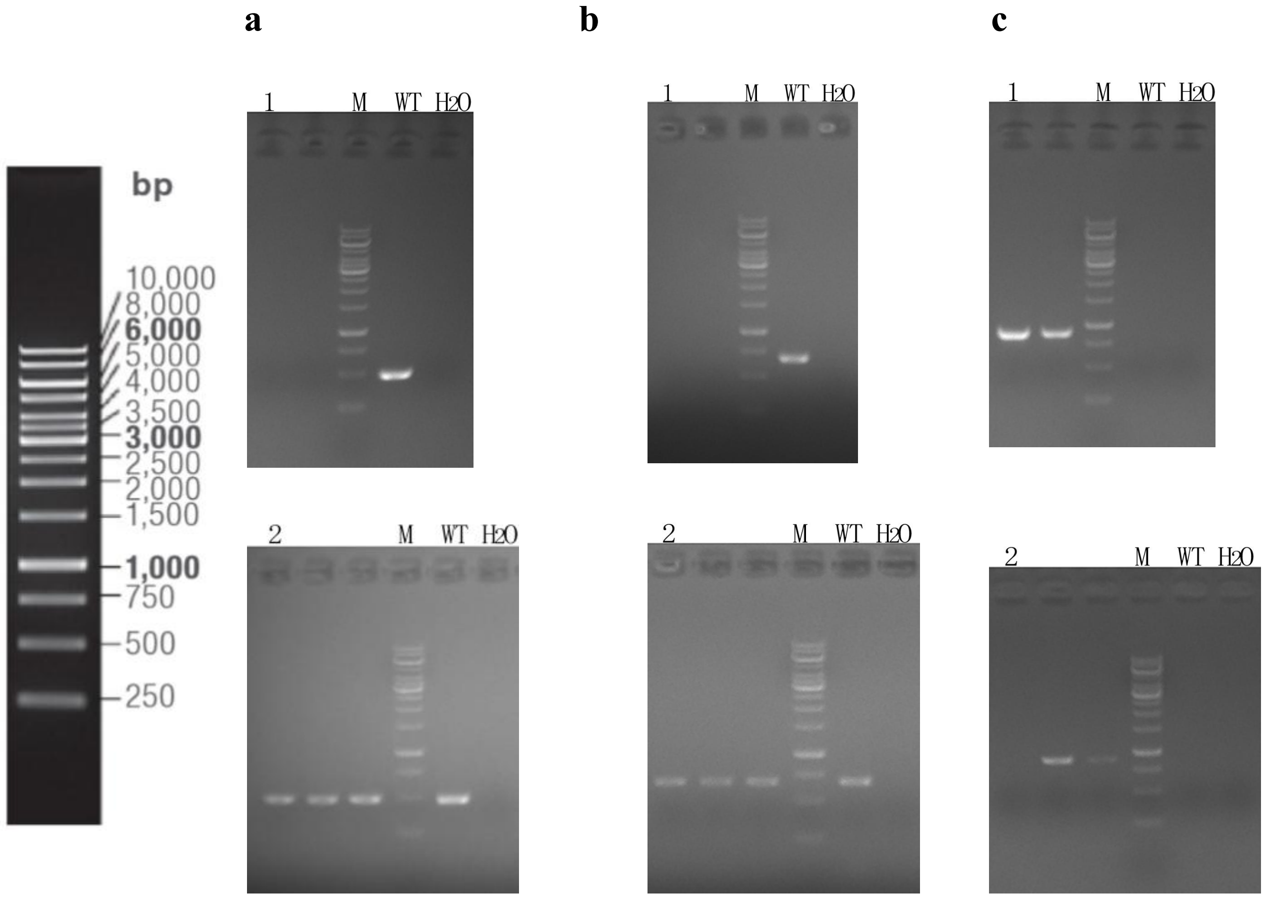

Fig. S3 PCR confirmation of HMGB2 knockout in NK-92 cells. **a** PCR amplification of genomic DNA from single-cell clones derived from the stable KO-HMGB2 NK-92 cell line using Primer 1, which targets the region around the gRNA-A1 sequence. **b** PCR amplification using Primer 2, which targets the gRNA-A2 sequence. **c** PCR amplification using Primer 3, which targets sequences flanking the knockout region. M, DNA marker; WT, wild-type NK-92 cells; H2O, negative control.
