# Supplementary material for: NK cell function down regulated by HMGB2 through ANGPT1/PI3K/AKT pathway and its effect on esophageal squamous carcinoma cells
Source: Front Immunol. 2025 Nov 7;16:1666199. doi: 10.3389/fimmu.2025.1666199 (PMC12634629; doi:10.3389/fimmu.2025.1666199)
Supplement: Supplementary file 2 [file DataSheet2.pdf]

Figure4.b siHMGB2

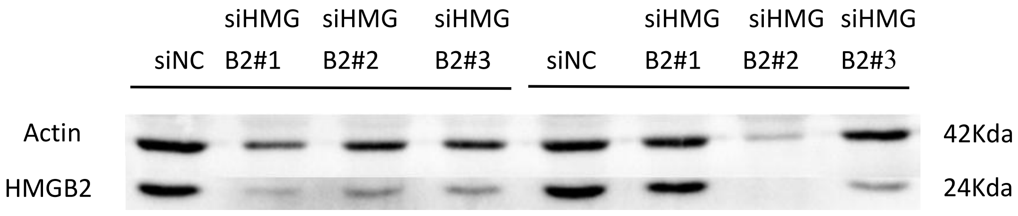

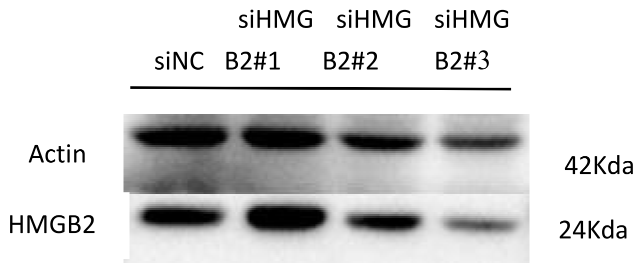

Figur5.b oeHMGB2

NK-92

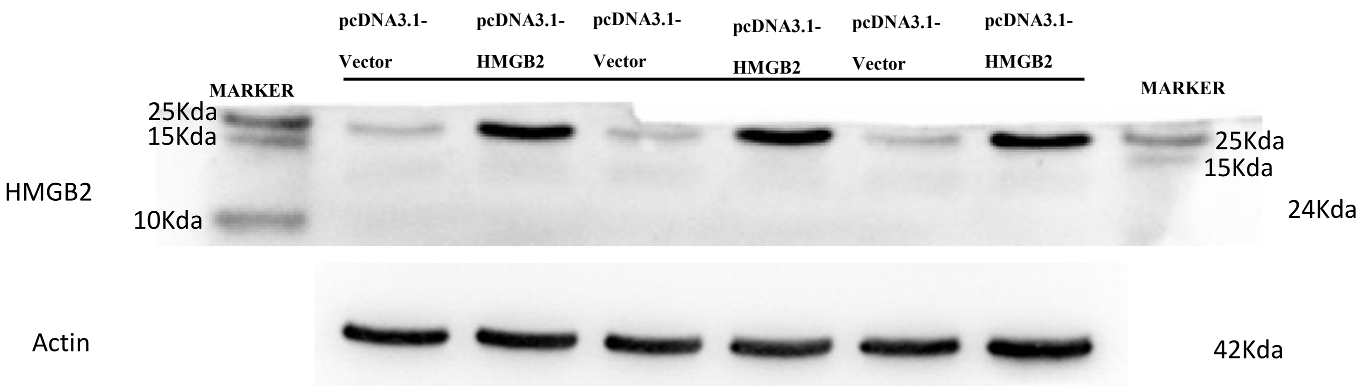

Figur7.c NK vs KO

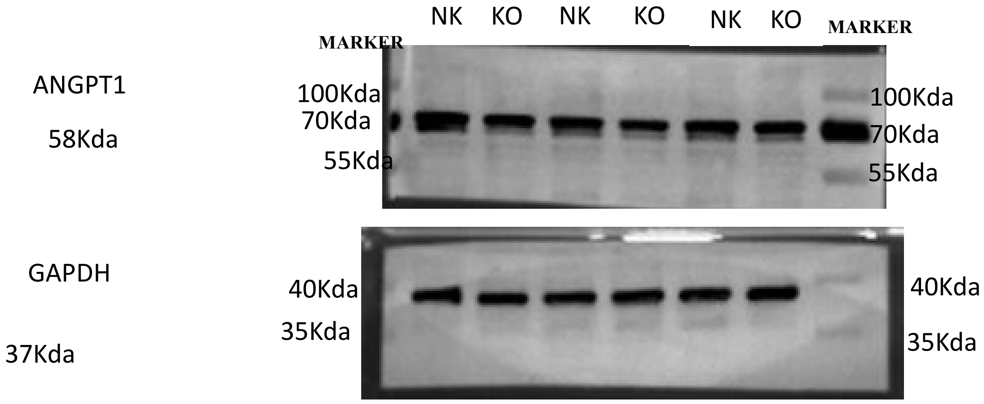

Figur7.d pi3k-akt

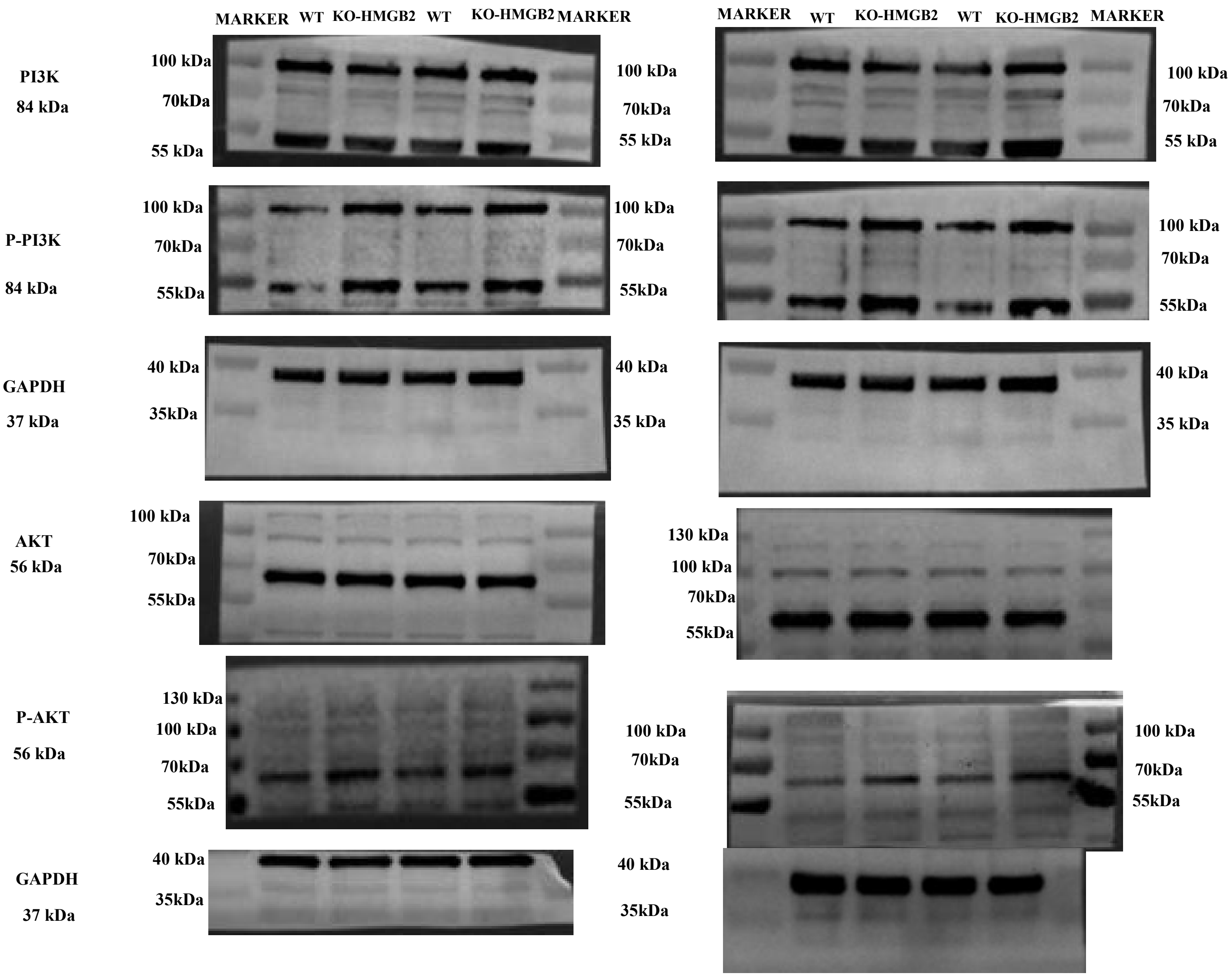

Figur6.b KO-HMGB2

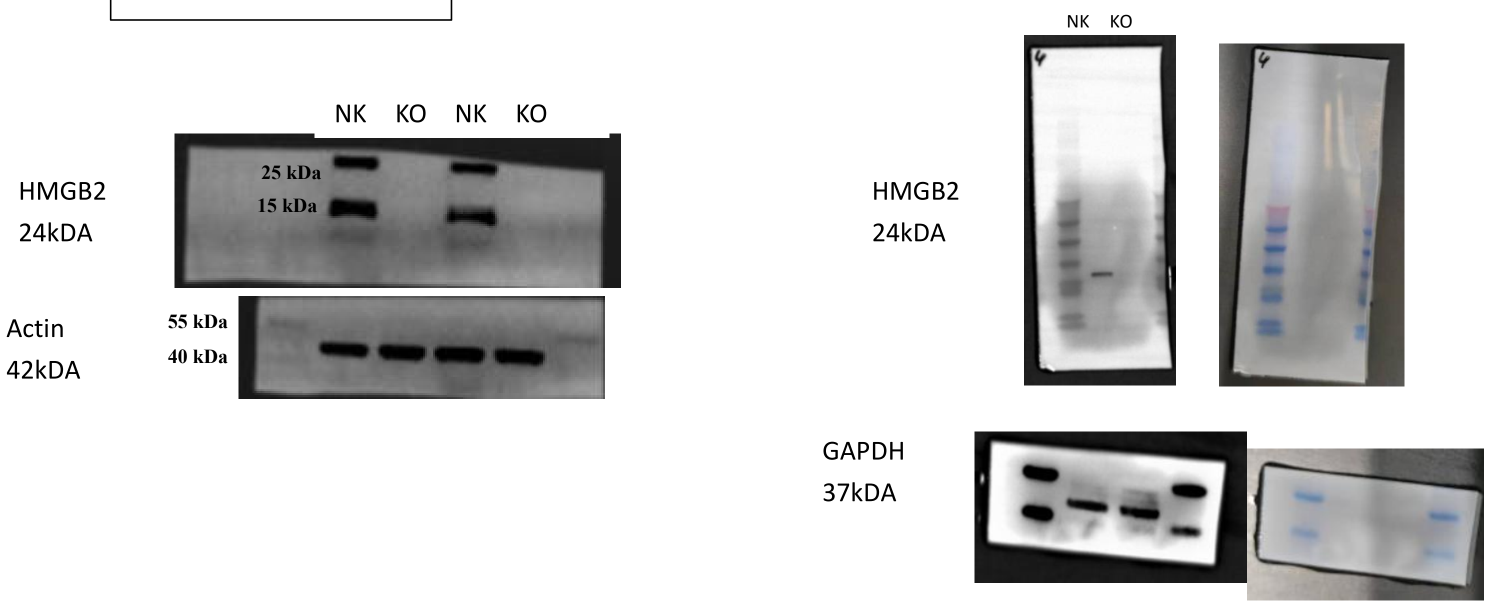

Marker：Three-color pre-stained protein Marker (11-245 kDa)

Figur7.e shANGPT1

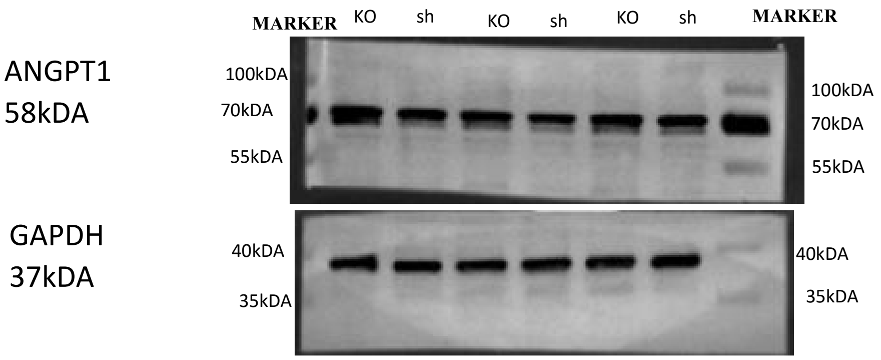

Figur7.f shANGPT1pi3k-akt

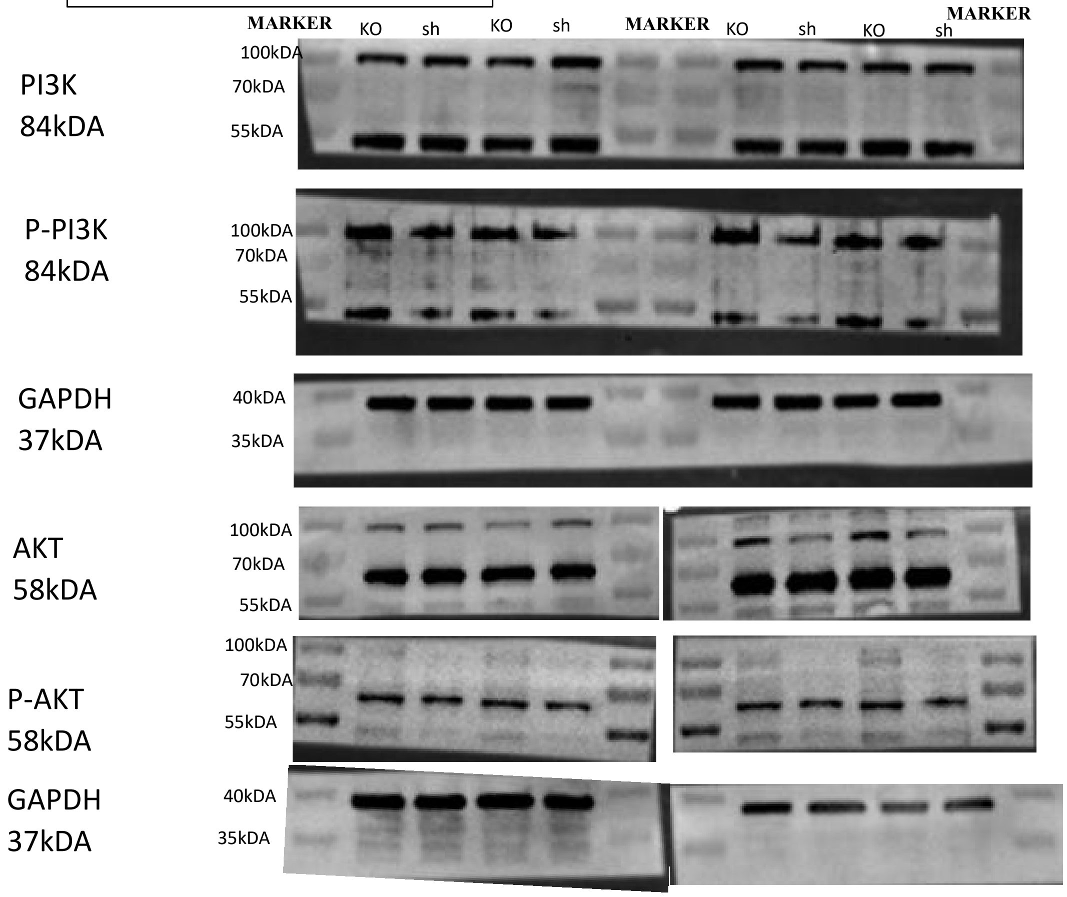

All WB experiments in this article, except where otherwise noted, used the Thermo Scientific PageRuler (26616) marker
