# Supplementary material for: NK cell function down regulated by HMGB2 through ANGPT1/PI3K/AKT pathway and its effect on esophageal squamous carcinoma cells
Source: Front Immunol. 2025 Nov 7;16:1666199. doi: 10.3389/fimmu.2025.1666199 (PMC12634629; doi:10.3389/fimmu.2025.1666199)
Supplement: Supplementary file 3 [file Table1.docx]

| Supplementary Table 1 Clinicopathological features in ESCC patients for RNA-seq and MS | |  |
| --- | --- | --- |
| Characteristics | ESCC(n=5) | |
| Age(year) |  | |
| <60 | **1** | |
| ≥60 | **4** | |
| Gender |  | |
| Female | **2** | |
| male | **3** | |
| Tumor size(cm) |  | |
| ≤4.0 | **4** | |
| >4.0 | **1** | |
| Pathological N |  | |
| N0 | **3** | |
| N1 | **2** | |
| Pathological T |  | |
| T1/T2 | **2** | |
| T3/T4 | **3** | |
| Stage |  | |
| I-IIA | **1** | |
| IIB-VI | **4** | |
