# Supplementary material for: NK cell function down regulated by HMGB2 through ANGPT1/PI3K/AKT pathway and its effect on esophageal squamous carcinoma cells
Source: Front Immunol. 2025 Nov 7;16:1666199. doi: 10.3389/fimmu.2025.1666199 (PMC12634629; doi:10.3389/fimmu.2025.1666199)
Supplement: Supplementary file 4 [file Table2.docx]

| Genes | Forward primer | Reverse primer |
| --- | --- | --- |
| β-Actin | 5’-GAGCTACGAGCTGCCTGACG-3’ | 5’- GTAGTTTCGTGGATGCCACAG-3’ |
| HMGB2 | 5’-GGTGAAATGTGGTCTGAGCAGTC-3’ | 5’-CCTGCTTCACTTTTGCCCTTGG-3’ |
| ANGPT1 | 5’-AGCGCCGAAGTCCAGAAAAC-3’ | 5’-TACTCTCACGACAGTTGCCAT-3’ |
| NKp46 | 5’-TGCCGTCTAGACACTGCAAC-3’ | 5’-CCAAAACATCGGTATGTCCC-3’ |
| NKG2D | 5’-TGGATTCGTGGTCGGAGGTCTC -3’ | 5’-TTGCTTTTGACTACTGGACATCTTTGC-3’ |
| NKp44 | 5’-TCACAGCCACAGAACTCCAC -3’ | 5’-CCTGAGCTCCATCATGGTTT-3’ |
| CD107a | 5’-CTCTGTGGACAAGTACAACGT-3’ | 5’-GTTGATGTTGAGAAGCCTTGTC -3’ |
